# In situ microcosm remediation of polyaromatic hydrocarbons: influence and effectiveness of Nano-Zero Valent Iron and activated carbon

**DOI:** 10.1007/s11356-022-22408-y

**Published:** 2022-08-09

**Authors:** Luisa Albarano, Maria Toscanesi, Marco Trifuoggi, Marco Guida, Giusy Lofrano, Giovanni Libralato

**Affiliations:** 1 Department of Biology, University of Naples Federico II, Via Vicinale Cupa Cintia 26, 80126 Naples, Italy; 2 Department of Marine Biotechnology, Stazione Zoologica Anton Dohrn, Villa Comunale, 80121 Naples, Italy; 3 Dipartimento Di Scienze Chimiche, Università Degli Studi Di Napoli Federico II, Via Vicinale Cupa Cintia 26, 80126 Naples, Italy; 4 Department of Movement, Human and Health Sciences, University of Rome “Foro Italico”, Rome, Italy

**Keywords:** Crustacean, Restoring methods, Nanomaterials, Toxicity, Genotoxicity

## Abstract

Nano-zero-valent iron (nZVI) and activated carbon (AC) addition are ongoing techniques for the remediation of hydrophobic organic compound-contaminated sediment and water, but with still unexplored eco(toxico)logical implications, especially when applied in situ. In this study, we investigated AC and nZVI as remediation methods for marine contaminated sediment and water, including chemical and toxicity (*Artemia franciscana* survival and genotoxicity) surveys. The removal efficiency of AC and nZVI (about 99%) was similar in both sediment and seawater, while the survival of nauplii and adults was mainly impacted by nZVI than AC. At the molecular level, the nZVI-addition induced down-regulation in the expression of two stress and one developmental genes, whereas AC was able to up-regulated only one gene involved in stress response. Results suggested that the use of AC is safer than nZVI that requires further investigation and potential optimization to reduce secondary undesired effects.

**Graphical abstract:**

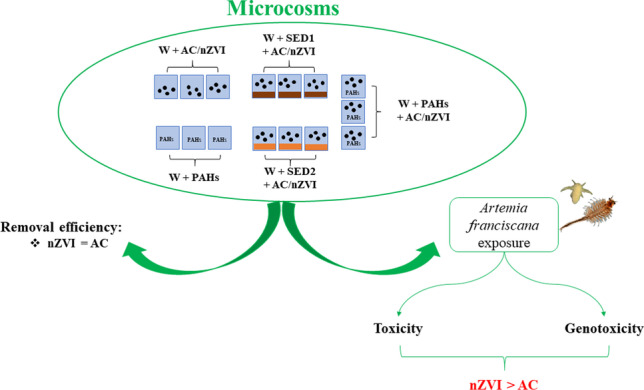

**Supplementary Information:**

The online version contains supplementary material available at 10.1007/s11356-022-22408-y.

## Introduction

Gulf of Naples extends along the coasts of the central to southern and Tyrrhenian Sea. Over the past few years, high concentrations of heavy metals (especially Cd, Cu, Zn, Cr, Ni, As), polycyclic aromatic hydrocarbons (PAHs), polychlorinated biphenyls (PCBs), and total hydrocarbons (HC) both in sediment and water from the stretch of sea have been reported by different studies (Montuori and Triassi 2012; Montuori et al. 2013; Arienzo et al. 2017, 2019; Trifuoggi et al. 2017; Morroni et al. 2020). In the northern sector, the Gulf was characterized by the past activity of ILVA plant of Bagnoli (the second largest integrated steel plant in Italy) (Arienzo et al. 2017, 2019; Trifuoggi et al. 2017). In the south-western sector site, the Gulf suffers by the presence of the Sarno River mouth (defined as “the most polluted river in Europe”) (Lofrano et al. 2016). The main causes of pollution of the Sarno River are the massive use of fertilizers and pesticides in agriculture and the industrial development. Among the substances mentioned above, the PAHs are certainly the pollutants most drained from the river in the Gulf (Montuori and Triassi 2012; Montuori et al. 2013).

PAHs are a well-known group of environmental pollutants predominantly generated by anthropogenic activities (about 99% of PAHs in the sediment are of anthropogenic origin) (Medeiros and Caruso Bícego 2004; Pedrazzani et al. 2019). The evidence of their genotoxicity and carcinogenicity for animal species is already available, and epidemiological studies demonstrated a correlation between PAHs exposure and cancer incidence. Benzo[a]pyrene (BaP) has been classified by the International Agency for research on cancer (IARC) as the prototypical carcinogenic and genotoxic PAH due to its adverse and toxic effects on various cells and tissues, reproduction, development, and immune system of animals (Knafla et al. 2006). Taking into consideration the high risk, various remediation activities have been implemented in recent years to reduce or removal toxic compounds using different chemical, thermal, and biological methods both in situ and ex situ treatments (Gomes et al. 2013; Lofrano et al. 2016). Among the most commonly used methods, there are chemical techniques, such as activated carbon (AC), nanozerovalent iron (nZVI), apatite (A), organoclay (OC), and zeolite (Z), which could negatively affect the resident biota, especially when applied in situ (Chen et al. 2016; Lofrano et al. 2016; De Gisi et al. 2017; Libralato et al. 2018; Albarano et al. 2020). Albarano et al. (2021a) evidenced that nZVI and AC were the most viable methods, even though preliminary results suggested a greater toxicity of nZVI. Activated carbon is a substance with high carbon content and porosity enabling to absorb different compounds (Rakowska et al. 2012; Li et al. 2020). Published data evidenced that the concentration of PAHs decreased in sediment after AC treatment ranging from 50 to 100% and being dependent on the quantity of amendments added (Hale et al. 2010; Bussan et al. 2016; Abel and Akkanen 2019). The nanozerovalent iron is characterized by a structure where the nucleus consists of a zerovalent and a mixed valent oxide shell of Fe^2+^ and Fe^3+^ that is formed as a result of oxidation of the core shell. The core–shell structure can provide to nZVI unique reactive surfaces with adsorption and transformation of contaminants via reductive or oxidative pathways (Li et al. 2021). Nanozerovalent iron is fairly reactive in water and possess excellent electron donating properties which makes it a versatile remediation material (Mukherjee et al. 2016), being quite widespread also due to its simplicity and cost effectiveness (100 €/kg) (Corsi et al. 2018; Ken and Sinha 2020; Zafar et al. 2021). The removal efficiency of organic compounds ranged from 90 to 99.8% in aqueous solutions after nZVI addition (Kim et al. 2010; Liu et al. 2012; Wu et al. 2020; Li et al. 2021).

The aim of this research was to investigate the restoration of PAHs contaminated seawater and marine sediment by nZVI and AC using microcosm experiments. Two natural sediment samples (i.e., with low and high pollution levels) were collected following a known pollution gradient (Montuori and Triassi 2012; Montuori et al. 2013; Arienzo et al. 2017, 2019; Morroni et al. 2020). The concentrations of PAHs in aqueous solution were chosen on the total PAHs concentration detected in the most polluted sediment. Specifically, AC and nZVI (i.e., 3% dry weight sediment) were separately added to all experimental conditions. Chemical analyses were conducted on water and sediment samples under the following conditions: just after adding the related amendment (T_0_), 3 h (T_1_), 6 h (T_2_), 21 h (T_3_), 24 h (T_4_), 72 h (T_5_), and 21 days (T_6_), to evaluate the efficiency of removal of AC and nZVI. Moreover, the potential negative impact of treatments was evaluated on embryos and adults of the branchiopod crustacean *Artemia franciscana* Kellog 1906, considering embryotoxicity, lethality, and genotoxicity. The crustacean *A. franciscana* is considered a good model species to investigate the ecotoxicological response of marine invertebrates to environmental pollutants (Libralato et al. 2007; Libralato 2014; Albarano et al. 2022). The greatest advantage of the species is that nauplii can be hatched as needed from commercially available durable cysts. Moreover, their small body size allows to conduct the tests in small beakers or plates; the embryo grows rapidly in laboratory conditions, and finally, they have adaptability to a wide range of salinities (5–300 g/L) and temperatures (6–40 °C) (Manfra et al. 2016). To best understand how *A. franciscana* protects itself from the stress caused by remediation methods, the gene pathways involved in stress response and development were evaluated (Chen et al. 2009; Albarano et al. 2022). Specifically, nauplii and adults were exposed to 100% not diluted aqueous solutions collected from all conditions at the end of experiment (T_6_).After 48 h of exposure, the effect on several key genes involved in stress response (*hsp26*, *hsp60*, *hsp70*, *COXI*, and *COXIII*) was assessed. In addition, the impact on developmental genes (*HAD*-like, *tcp*, *UCP2*, and *CDC48*) was also evaluated for nauplii.

## Materials and methods

### Sediment collection and chemical characterization

**Fig. 1:**
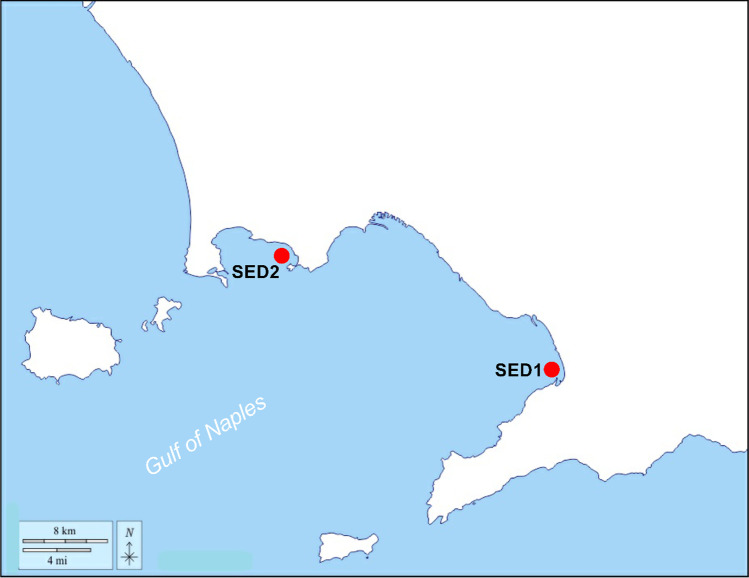
Map of the Gulf of Naples with location of sediment collection sites SED1 and SED2

Sediments were sampled with a Van Veen grab between January 2020 and February 202, at two sites in the Gulf of Naples (Tyrrhenian Sea, Italy; Fig. 1). Sediments were collected from Sarno River mouth at 1500 m South (namely SED1; 40°42′41.38" N and 14°28′45.14" E) and from Bagnoli Bay (namely SED2; 40°48′54.8" N and 14°09′44.2" E). These samples were chosen, following previous environmental characterizations of the Sarno River (Montuori and Triassi 2012; Montuori et al. 2013) and Bagnoli Bay (Arienzo et al. 2017, 2019; Morroni et al. 2020). After collection, sediment samples were stored at − 20 °C for chemical analyses, until analyzed for grain-size distribution (gravel, sand, silt, and clay) and PAHs. For grain size analysis, an amount of 50 g per sample was treated with 10% H_2_O_2_ and distilled water (2:8) for 48 h at room temperature in order to remove salts and organic matter. After drying (24 h at 105 °C), sediment fractions were mechanically separated with multiple vibrating sieves (Ro-Tap Particle Separator, Giuliani, Haver & Boecker Oelde Germany) with a 63 -m mesh to distinguish between sandy and silt–clay fractions (Albarano et al. 2021b; Danovaro 2010; Danovaro et al. 2008). Each fraction was weighted separately. Gain size data were analyzed with GradiStat software version 8.0 (Blott and Pye 2001) and expressed as a percentage of the total dry weight.

The determination of PAHs in sediment was performed by extracting 5 g dry sediment extracted with acetone/n-hexane 1:1 v/v (10 mL), using an ultrasonic distruptor (Brason,US). The extract was concentrated to 1 mL in multivap under nitrogen flow (Multivap, LabTech, Italy). Ten mircroliters of a 1 mg/L solution of internal standard (mixture of deuterated PAHs) was added to the extract, and injected to a gas-chromatography mass-spectrometry (GC–MS) (MS-TQ8030-Shimadzu, Japan). For sediment samples, the limit of detection (LOD) and limit of quantification (LOQ) values were 0.16 and 0.1 μg/kg, respectively. Data quality was ensured by certified reference materials (ERM-CA100 (European Commission)) and the recovery percentage was 70–110% (Arienzo et al. 2017; Carotenuto et al. 2020; Albarano et al. 2021b). The characteristics of the sediments and initial concentrations of PAHs are summarized in Fig. S1 and Table S1, respectively.

### Amendments

The remediation methods evaluated in the present study were nZVI and AC. NANOFER-Star-Zero-Valent-iron (air-stable powder of FeNPs stabilized by inorganic stabilizers; nZVI) was purchased from NANOIRON Future Technology (Židlochovice Czech Republic), with particle sizes smaller than 100 nm. Activated carbon (air-stable powder; AC) was purchased from J.T. Baker® (Deventer, Holland) with particle sizes smaller than 100 nm.

### Experimental design

Two experiments were performed to determine changes in sediment and seawater PAHs concentrations and the concentration of available PAHs after a period of remediation with activated carbon and nano-zero-valent iron. The conditions of experiments included five scenarios: (i) negative control – synthetic seawater (SSW, prepared in according to ISO 10253/16) plus amendments (W + AC or W + nZVI); (ii) positive control – seawater spiked with PAHs (1800 μg/L, nominal; according to total PAHs concentration in Bagnoli sediment reported in Table S1) (W + PAHs) to check the loss of PAHs by evaporation; (iii) seawater spiked with PAHs + amendments (W + PAHs + AC or W + PAHs + nZVI); (iv) seawater + sediment collected from Sarno River (SED1) with amendments (W + SED1 + AC or W + SED1 + nZVI); and (v) seawater + sediment collected from Bagnoli Bay (SED2) plus amendments (W + SED2 + AC or nZVI).

Each of the 15 testing microcosms, located at the University of Naples, Federico II, was characterized by an independent and closed seawater system (glass bottle). Each bottle (500 mL; the size was chosen according to amount of sediment necessary for the specific test run) was filled with 50 g of sediment and topped with 150 mL of synthetic seawater respecting a ratio of 1:4 (sediment: seawater). An amount of 1.7 g of AC and nZVI (3% sediment dry wt) according to Brändli et al. (2009) and Choi et al. (2009) was added to all experimental conditions with exception of positive control (W + PAHs). These microcosms were shaken on orbital shaker (120 rpm) at 20 ± 1 °C in darkness condition for 21 days. All experiments were carried out in triplicates.

To evaluate the changes in PAHs concentration in sediment and seawater, 20 mL of liquid sample and 5 g of sediment were withdrawn using a glass pipet after the addition of amendments (T_0_), 3 h (T_1_), 6 h (T_2_), 21 h (T_3_), 24 h (T_4_), 72 h (T_5_), and 21 days (T_6_).

For PAHs concentration after nZVI remediation, seawater and sediment samples were extracted according to (Albarano et al. 2021b; Arienzo et al. 2017; Carotenuto et al. 2020). For PAHs analyses after AC restoring, seawater samples were extracted by a solid-phase extraction (SPE): 50 ml of water was filtered and preconcentrated on a C18 disk (ENVI, -18 DSK SPE Disk, diam. 47 mm). The analytes were eluted with a solution of 1:1 dichloromethane and n-hexane. The extract was then concentrated to 1 mL in Multivap under nitrogen flow. The determination in the sediment was performed by considering 5 g of dry sediment extracted with carbon sulfide v/v (10 mL), using an ultrasonic disruptor (Brason, US). The extract was concentrated to 1 mL in Multivap under nitrogen flow (Multivap, LabTech, Italy). A total of 10 μL of a 1 mg/L solution of internal standard (mixture of deuterated PAHs) was added to the sediment and seawater extracts and injected to a gas chromatography–mass spectrometry (GC–MS) (MS-TQ8030-Shimadzu, Japan).

### Acute toxicity test

Acute toxicity tests using both *A. franciscana* nauplii and adults were performed according to standard methods (CNR 2003) using lethality as an endpoint. Effects were measured after 48 h of exposure for both adults and nauplii up to the third instar (corresponding to 48-h-old specimen that are considered as the most sensitive stage). Certified dehydrated cysts of brine shrimp *A. franciscana* (AF/F2005) were purchased from the company Ecotox LDS (Gallarate, Italy). Hatching of the cysts was obtained by incubating 100 mg of cysts in glass Petri dishes containing seawater prepared by dissolving 36 g of Instant Ocean® salt in deionized water, stirred for 24 h under aeration, and then filtered through 0.45-μm Millipore cellulose filters. Newly hatched brine shrimp larvae (Instar I nauplius stage) were separated from unhatched cysts and transferred, taking advantage of phototactic movements, into new glass Petri dishes with synthetic seawater (SSW) prepared according to ISO 10253/16 (2016).

Ten nauplii and five adults were exposed to increasing percentage concentrations (0%, 6.25%, 12.5%, 25%, 50%, and 100%) of aqueous solutions, collected from all experimental conditions using a glass pipet at T_0_, T_1_, T_2_, T_3_, T_4_, T_5_, and T_6_. The plates were kept at 25 ± 1 °C with salinity 35 ppm for 48 h in a light regime of 16:8-h light:dark, without providing any food. At 48 h, the number of nauplii and adults (which were motionless for 10 s) was counted under a stereomicroscope (Leica EZ4 HD) to calculate the lethality. Tests were considered valid when mortality in negative controls was < 10% after 48 h of exposure. All experiments were performed in triplicates.

### Organisms exposures for RNA extraction, cDNA synthesis and real-time q-PCR

Two hundred nauplii and ten adults of *A. franciscana* were exposed to 100% non-diluted aqueous solutions collected from all conditions at the end of experiment (T_6_). All experiments were performed in triplicates. Samples were collected after 48 h of exposure by centrifugation at 4000* g* for 15 min in a swing out rotor at 4 °C in a 2-mL tube, kept on ice, and were further homogenized in TRIzol (Invitrogen, Paisley, UK) using a TissueLyser II (Qiagen, Valencia, CA, USA) and steal beads of 7-mm diameter (Qiagen, Valencia, CA, USA). Total RNA was extracted and purified using Direct-zolTM RNA Miniprep Plus Kit (ZYMO RESEARCH). The amount of total RNA extracted was estimated by the absorbance at 260 nm and the purity by 260/280 and 260/230-nm ratios, using a NanoDrop spectrophotometer 2000 (Thermo Scientific Inc., Waltham, MA USA), to exclude the presence of proteins, phenol, and other contaminants (Riesgo et al. 2012). For each sample, 1000 ng of total RNA was retrotranscribed with an iScript™ cDNA Synthesis kit (Bio-Rad, Milan, Italy), following the manufacturer’s instructions. The variations in the expression of five genes involved in stress response (*hsp26*, *hsp60*, *hsp70*, *COXI*, and *COXIII* (Chen et al. 2009); see Supplementary Fig. S2) were evaluated for adults. For nauplii, the variations in the expression of four other genes involved in developmental and differentiation processes (*HAD*-like, *tcp*, *UCP2*, and *CDC48*, Chen et al. 2009) were also tested (Supplementary Fig. S2). Undiluted cDNA was used as a template in a reaction containing a final concentration of 0.3 mM for each primer and 1 × SensiFAST™ SYBR Green master mix (total volume of 10 μL) (Meridiana Bioline). PCR amplifications were performed in AriaMx real-time PCR instrument (Agilent Technologies, Inc.), according to the manufacturer’s instructions system thermal cycler, using the following thermal profile: 95 °C for 10 min, one cycle for cDNA denaturation; 95 °C for 15 s and 60 °C for 1 min, 40 cycles for amplification; 95 °C for 15 s, one cycle for final elongation; one cycle for melting curve analysis (from 60 to 95 °C) to verify the presence of a single product. Each assay included a no-template control for each primer pair. To capture intra-assay variability, all real-time qPCR reactions were carried out in triplicate. Fluorescence was measured using Agilent Aria 1.7 software (Agilent Technologies, Inc.). The relative expression ratios were calculated according to (Pfaffl 2001; Pfaffl et al. 2002) using REST software (Version No., Relative Expression Software Tool, Weihenstephan, Germany). The expression of each gene was analysed and internally normalized against *GAPDH* (Chen et al. 2009) using REST software (Relative Expression Software Tool, Weihenstephan, Germany) based on the Pfaffl method (Pfaffl 2001; Pfaffl et al. 2002). Relative expression ratios above 1.5 were considered as significant.

### Statistical analyses

Toxicity data were reported as “mean ± one standard deviation (SD).” Data were checked for normality using the Shapiro–Wilk’s (S-W) test (*p* < 0.05). The statistical significance of differences among different percentage of treatments and control was checked by two-way ANOVA followed by Tukey’s test for multiple comparisons (GraphPad Prism Software version 8.02 for Windows, GraphPad Software, La Jolla, California, USA, www.graphpad.com). *P*-values < 0.05 were considered statistically significant.

## Results

### Effect of nZVI and AC dosage on PAHs removal

As reported in Supplementary Fig. S3, results demonstrated that the nZVI addition induced high PAHs degradation both in sediment and seawater. When considered W + PAHs + nZVI condition, already after 21 h (T_3_) of treatment, the degradation of pollutants was total (Supplementary Fig. S3). Since the loss of total PAHs by evaporation at T_3_ was only 20%, this result can be considered completely due to the treatment with nZVI. Considering the aqueous solution of SED1 and SED2 (W + SED1 + nZVI and W + SED2 + nZVI, respectively), no PAHs have been detected at all studied times (from T_0_ to T_6_) with only T_3_ and T_4_ exception of W + SED1 + nZVI (see also Supplementary Fig. S3), where little concentrations of 9 and 8 μg/L have been shown, respectively.

In the case of Sarno sediment (SED1), the removal of hydrocarbons was equal to 100% already after 3 h of treatment (T_1_), whereas taking in the consideration the Bagnoli sediment (SED2), approximately 26 μg/kg still remained at the end of experiment (after 21 days, T_6_) (Supplementary Fig. S3). In assessing the removal of individual compounds from SED2 (Supplementary Fig. S4), the results showed that this amendment was not able to completely remove fluoranthene, pyrene, benzo[b]fluoranthene, benzo[a]anthracene, Indeno(1,2,3-cd)pyrene, and benzo[a]pyrene.

The addition of 3% AC reduced the aqueous concentration of PAHs, with pollutants concentrations below the LOD already after 21 h (T_3_) of treatment (Supplementary Fig. S5). Also in this case, since the loss of total PAHs by evaporation at T_3_ was only 20%, this result can be associated to the AC treatment. Moreover, considering the aqueous solution of SED1 and SED2 (W + SED1 + AC and W + SED2 + AC, respectively), no PAHs were detected at all studied time (from T_0_ to T_6_; see also Supplementary Fig. S5). The results obtained from sediment treatments with AC were almost similar to those obtained for nZVI. In fact, total removal of PAHs was found for SED1, whereas a residue of about 25.7 μg/kg was still measured in the sediment of Bagnoli (SED2) from T_1_ to the end of experiment (after 21 days, T_6_) (Supplementary Fig. S5). In assessing the removal of individual compounds from SED2 (Supplementary Fig. S6), the results showed that this amendment was not able to completely remove the fluoranthene, benzo[a]anthracene, dibenzo[a,h]anthracene, Indeno[1,2,3-cd]pyrene, and benzo[a]pyrene. The removal efficiencies of two remediation methods demonstrated that they were much more efficient for PAHs removal from aqueous solutions than sediments (Table 1). The degradation efficiencies were 98.3% and 99.6% for total PAHs removal from aqueous solutions (W + PAHs + nZVI or AC; see Table 1), and 98.9% from Sarno sediment (SED1) plus amendments (SED1 + nZVI or AC). The percentage of total PAHs removal from Bagnoli sediment was 60.6% and 49.3% for AC and nZVI methods, respectively (Table 1).

**Table 1:** Removal efficiencies achieved with different remediation methods. *n.a.* not available, AC = activated carbon, nZVI = nano-Zero Valent Iron

	W + PAHs + AC (μg/L)			W + PAHs + nZVI (μg/L)		
	T_0_	T_6_	**Removal efficiency (%)**	T_0_	T_6_	**Removal efficiency (%)**
	**AC**			**nZVI**		
Acenaphthylene	0	0	n.a	0	0	n.a
Acenaphthene	0.1	0.1	0	157.7	0.25	99.8
Fluorene	10.6	0.1	99.1	176	0.25	99.9
Anthracene	6.35	0.1	98.4	13	0.25	98.1
Phenanthrene	0	0	n.a	0	0	n.a
Fluoranthene	6.89	0.1	98.5	88	0.25	99.7
Pyrene	7.63	0.1	98.7	32	0.25	99.2
Benzo[a]antracene	0	0	n.a	0	0	n.a
Chrysene	5.24	0.1	98.1	2.3	0.25	89.1
Benzo[b]fluorantene	0	0	n.a	0	0	n.a
Benzo[k]fluoranthene	3.68	0.1	97.3	1	0.25	75
Benzo[a]pyrene	0	0	n.a	0	0	n.a
Indeno[1,2,3-cd]pyrene	0	0	n.a	0	0	n.a
Dibenz[a,h]anthracene	0	0	n.a	0	0	n.a
Benzo[ghi]perylene	0	0	n.a	0	0	n.a
**Total PAHs**	**2.70**	**0.05**	**98.3**	**31.3**	**0.1**	**99.6**
	**SED1 + AC (μg/Kg)**			**SED1 + nZVI (μg/Kg)**		
Acenaphthylene	4.18	0.5	88.0	4.18	0.5	88.0
Acenaphthene	0	0	n.a	0	0	n.a
Fluorene	0	0	n.a	0	0	n.a
Anthracene	11.75	0.5	95.7	11.75	0.5	95.7
Phenanthrene	0	0	n.a	0	0	n.a
Fluoranthene	74.5	0.5	99.3	74.5	0.5	99.3
Pyrene	63.15	0.5	99.2	63.15	0.5	99.2
Benzo[a]antracene	56.97	0.5	99.1	56.97	0.5	99.1
Chrysene	59.96	0.5	99.2	59.96	0.5	99.2
Benzo[b0fluorantene	85.26	0.5	99.4	85.26	0.5	99.4
Benzo[k]fluoranthene	35.46	0.5	98.6	35.46	0.5	98.6
Benzo[a]pyrene	67.13	0.5	99.3	67.13	0.5	99.3
Indeno[1,2,3-cd]pyrene	51.59	0.5	99.0	51.59	0.5	99.0
Dibenz[a,h]anthracene	12.55	0.5	96.0	12.55	0.5	96.0
Benzo[ghi]perylene	44.82	0.5	98.9	44.82	0.5	98.9
**Total PAHs**	**37.8**	**0.4**	**98.9**	**37.8**	**0.4**	**98.9**
	**SED2 + AC (μg/Kg)**			**SED2 + nZVI (μg/Kg)**		
Acenaphthylene	6.5	2	69.2	15.6	8.39	46.2
Acenaphthene	2	2	0.0	2	0.47	76.5
Fluorene	2	2	0.0	2	0.96	52.0
Anthracene	85.2	11.2	86.9	19.7	15.04	23.7
Phenanthrene	35.4	10.4	70.6	11.4	4.87	57.3
Fluoranthene	125	54.6	56.3	130.8	77.61	40.7
Pyrene	146	35.6	75.6	101.2	60.75	40.0
Benzo[a]antracene	85.6	54.6	36.2	57.8	30.62	47.0
Chrysene	64.7	10.3	84.1	47.7	26.87	43.7
Benzo[b]fluorantene	32.4	5.6	82.7	112.6	45.45	59.6
Benzo[k]fluoranthene	78.9	26.8	66.0	42.4	21.92	48.3
Benzo[a]pyrene	65.4	35.6	45.6	81.1	35.73	55.9
Indeno[1,2,3-cd]pyrene	74.5	45.3	39.2	70.4	30.65	56.5
Dibenz[a,h]anthracene	104.3	85.6	17.9	13.4	4.53	66.2
Benzo[ghi]perylene	68.9	3.6	94.8	60.7	25.93	57.3
**Total PAHs**	**65.1**	**25.7**	**60.6**	**51.3**	**26.0**	**49.3**

### Toxicity effect of nZVI and AC on nauplii

**Fig. 2:**
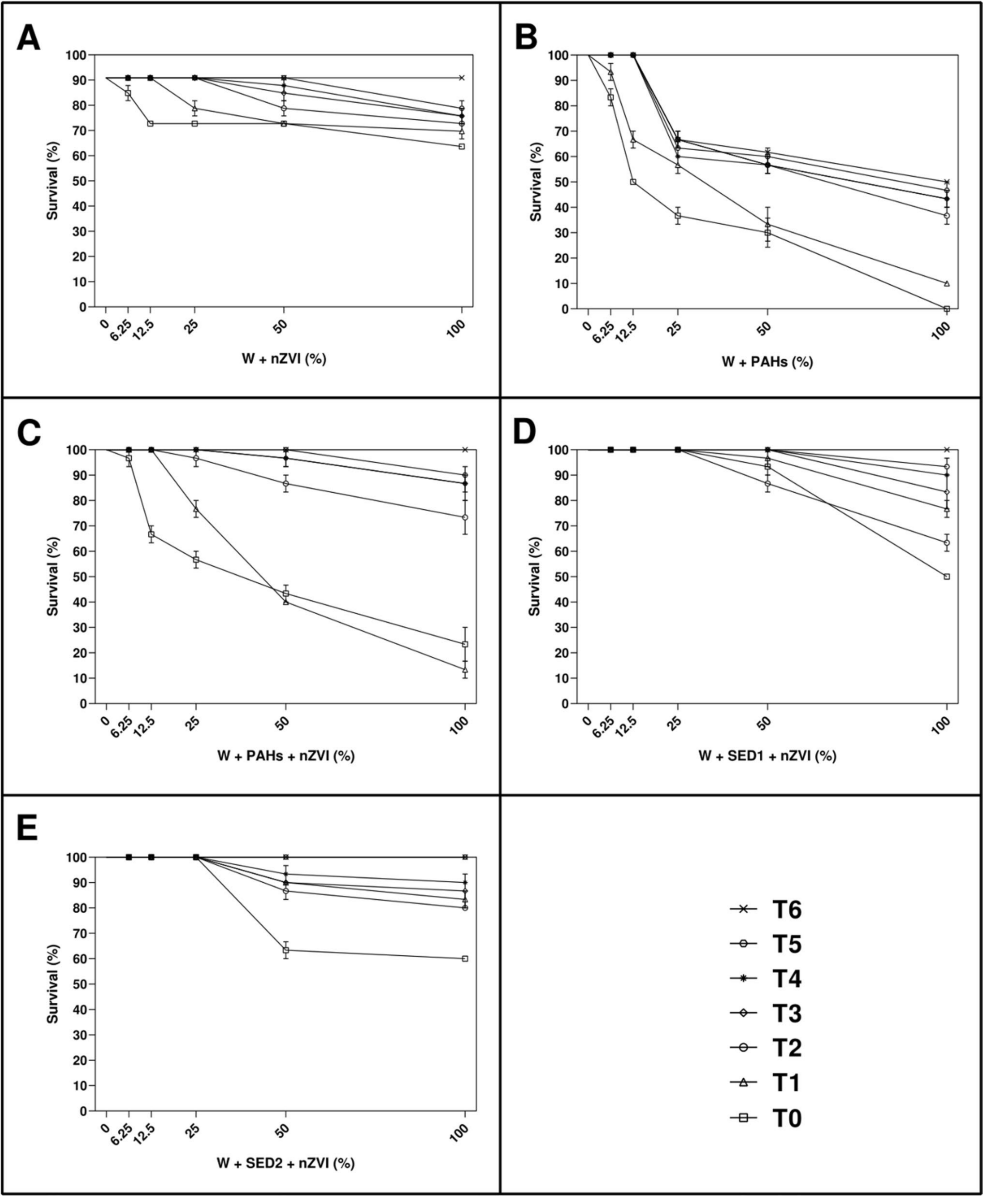
After 48 h, the percentage of surviving nauplii detected at seven time (T_0_ = after adding amendment; T_1_ = 3 h; T_2_ = 6 h; T_3_ = 21 h; T_4_ = 24 h; T_5_ = 72 h; and T_6_ = 21 days) both in control (0%) and treated samples with 6.25%, 12.5%, 25%, 50%, and 100% of **A** W + nZVI; **B** W + PAHs; **C** W + PAHs + nZVI; **D** W + SED1 + nZVI; and **E** W + SED1 + nZVI. Data are reported as mean ± standard deviation

As reported in Fig. 2, after 48 h of exposure to different percentage of aqueous solutions of all experimental conditions of nZVI, an increase of toxicity was observed at higher tested percentage, represented by 50% and 100%.

Considering W + nZVI condition at T_0_ (Fig. 2A), a little percentage of dead nauplii (about 6.6%) has been shown already at the lowest percentage (6.25%). These data were statistically significant with respect to the control (*p* < 0.05) and others used concentrations (*p* < 0.0001; see also Supplementary Table S2). At 100%, significant increase of toxicity (about 30%) respecting lower (0% and 12.5%; *p* < 0.0001 and *p* < 0.001, respectively) and higher (25% and 50%; *p* < 0.001) tested concentrations has been shown. Taking into consideration T_1_, at 25%, 50%, and 100% a significant decrease of survival (about 13%, 20%, and 23%, respectively) respecting lower concentrations (0%, 6.25% and 12.5%; *p* < 0.0001 (Supplementary Table S2) was detected. Moreover, the data reported at 25% were statistically significant respecting to 50% (*p* < 0.05) and 100% (*p* < 0.001). Considering T_2_, T_3_, and T_4_ only at 50% and 100%, a significant decrease of survival (about 10% and 20%, respectively) respects all lower concentrations (*p* < 0.0001) and among them (*p* < 0.001; Supplementary Table S2).

As reported Fig. 2B, W + PAH condition at T_0_ and T_1_ caused a little survival decrease (about 10%) already at 6.25%, which was statistically significant respecting to control (*p* < 0.0001) and other concentrations (*p* < 0.0001; Supplementary Table S2). At 25%, a significant decrease of about 50% respecting lower concentrations (*p* < 0.0001 (Supplementary Table S2)) and higher concentrations (50% and 100%; *p* < 0.0001) was detected. From T_2_ to T_6_, the results were similar (Fig. 2B). In fact, at 25%, a significant decrease of about 30% respecting lower concentrations (*p* < 0.0001 (Supplementary Table S2) and higher concentrations (50% and 100%; *p* < 0.0001) has been shown.

Considering W + PAHs + nZVI condition at T_0_ and T_1_ (Fig. 2C), a low percentage of dead nauplii (about 25%) was detected at 12.5%. At 100%, significant increase of toxicity (about 80%) respecting lower (0%, 6.25%, and 12.5%; *p* < 0.0001) and higher (25% and 50%; *p* < 0.0001) tested concentrations was measured (Supplementary Table S2). Considering T_2_, T_3_, T_4_, and T_5_ only at 50% and 100%, a significant decrease of survival (about 10%) respects all lower concentrations (*p* < 0.0001) and among them (*p* < 0.0001; Supplementary Table S2). At T_6_, no toxicity was found.

Take into the consideration W + SED1 + nZVI condition at T_0_, T_1_, and T_3_ (Fig. 2D), a low percentage of dead nauplii (about 10%) was showed at 50%. At 100%, significant increase of toxicity (about 20–30%) respecting lower (0%, 6.25% and 12.5%; *p* < 0.0001) and higher (25% and 50%; *p* < 0.0001) tested concentrations was showed (Supplementary Table S2). At T_4_, T_5_, and T_6_, no toxicity was detected.

Similar scenario can be described for W + SED2 + nZVI condition (Fig. 2E). In fact, from time 0 to time 4 only at 50% and 100%, a significant decrease of survival (about 10% and 20%, respectively) respects all lower concentrations (*p* < 0.0001). At T_5_ and T_6_, no toxicity was observed.

**Fig. 3:**
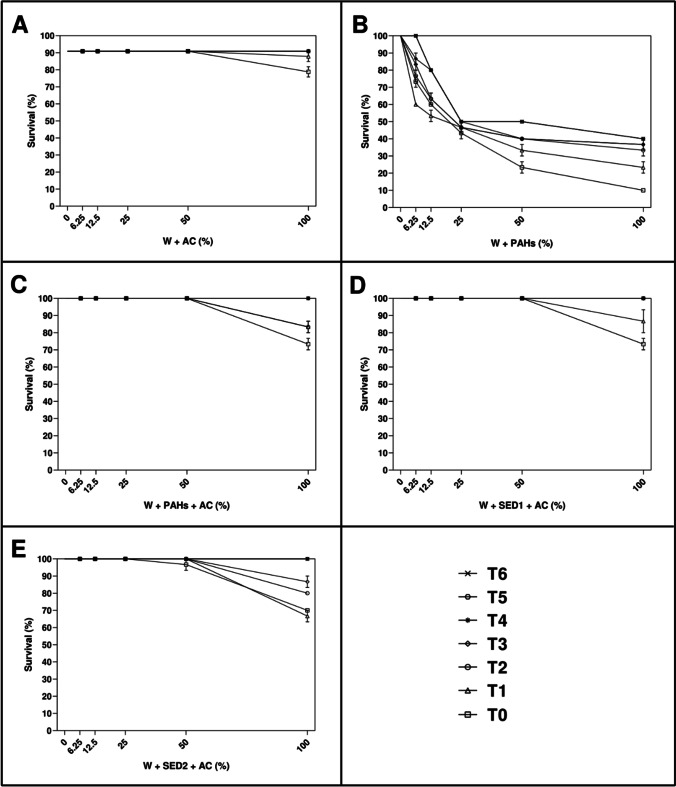
After 48 h, the percentage of surviving nauplii detected at seven time (T_0_ = after adding amendment; T_1_ = 3 h; T_2_ = 6 h; T_3_ = 21 h; T_4_ = 24 h; T_5_ = 72 h; and T_6_ = 21 days) both in control (0%) and treated samples with 6.25%, 12.5%, 25%, 50%, and 100% of **A** W + AC; **B** W + PAHs; **C** W + PAHs + AC; **D** W + SED1 + AC; and **E** W + SED1 + AC. Data are reported as mean ± standard deviation

As reported in Fig. 3, after 48 h of exposure to different percentage of aqueous solutions of all experimental conditions of AC, the scenario was a little different.

Considering W + AC condition at T_0_ and T_1_ (Fig. 3A), at 100%, significant percentage of dead nauplii (about 10–20%) was detected in comparison with the other concentrations (0%, 6.25%, 12.5%, 25%, and 50%; *p* < 0.0001, see also Supplementary Table S3). Taking into consideration T_2_, T_3_, T_4_, T_5_, and T_6_, no toxicity was detected. At the same manner, take into consideration W + PAHs + AC and W + SED1 + PAHs (Fig. 3C and D), only at 100%, significant percentage of dead nauplii (about 10–20%) was observed with respect to the other concentrations (0%, 6.25%, 12.5%, 25%, and 50%; *p* < 0.0001, see also Supplementary Table S3).

On considering W + SED2 + AC (Fig. 3E), from T_0_ to T_3_, at 100%, significant percentage of dead nauplii (from about 10 to 30%) was observed with respect to other concentrations (*p* < 0.0001, see also Supplementary Table S3). At T_4_, T_5_, and T_6_, no decrease of survived nauplii was observed.

### Toxicity effect of nZVI and AC on adults

**Fig. 4:**
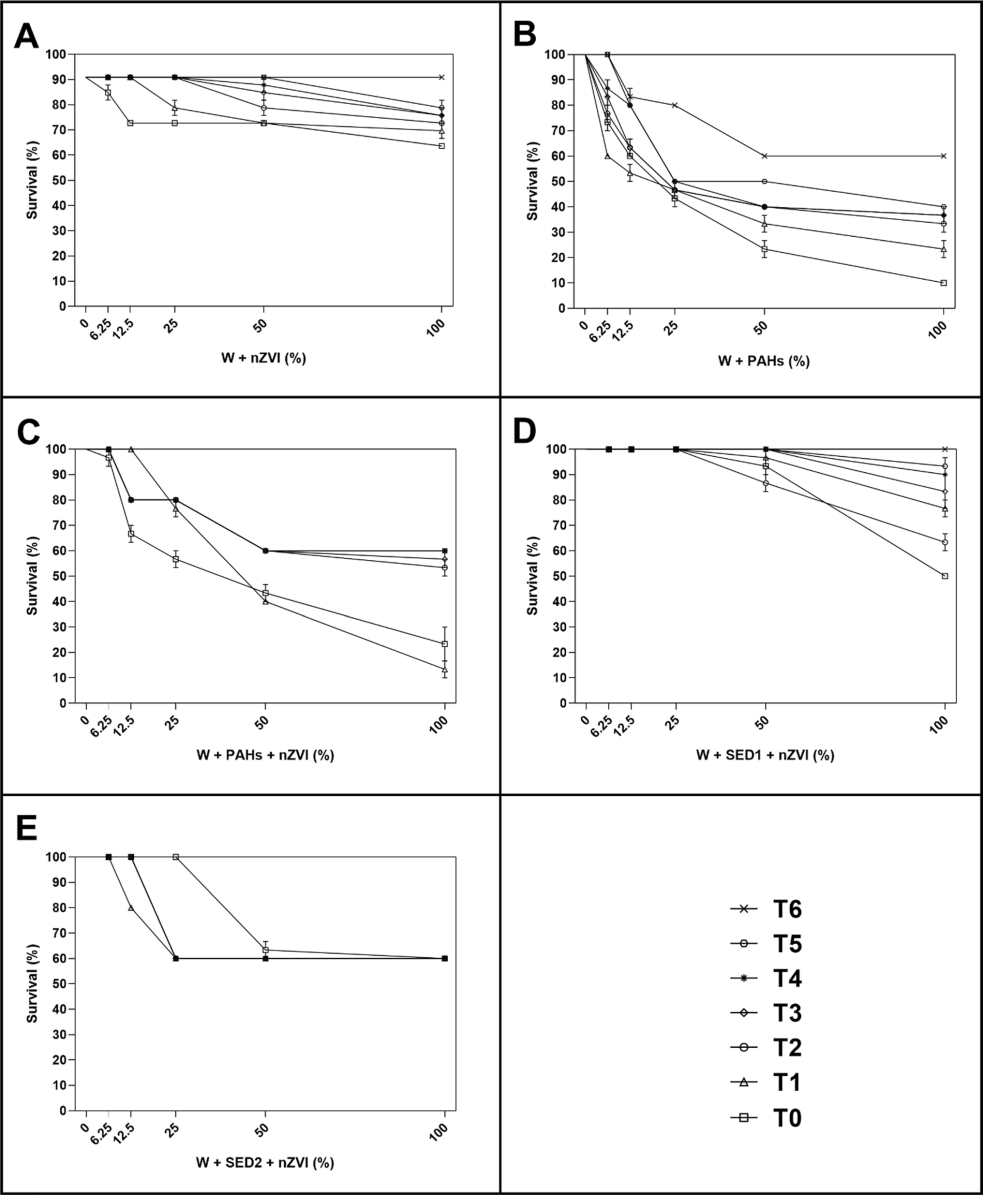
After 48 h, the percentage of surviving adults detected at seven time (T_0_ = after adding amendment; T_1_ = 3 h; T_2_ = 6 h; T_3_ = 21 h; T_4_ = 24 h; T_5_ = 72 h; and T_6_ = 21 days) both in control (0%) and treated samples with 6.25%, 12.5%, 25%, 50%, and 100% of **A** W + nZVI; **B** W + PAHs; **C** W + PAHs + nZVI; **D** W + SED1 + nZVI; and **E** W + SED1 + nZVI. Data are reported as mean ± standard deviation

On evaluating the toxicity on adults, the scenario was found to be almost similar to that presented for nauplii. As reported in Fig. 4, after 48 h of exposure to different percentage of aqueous solutions of all experimental conditions of nZVI, an increase of toxicity was observed at higher tested percentage, represented by 50% and 100%.

Considering W + nZVI condition at T_0_ (Fig. 4A), a little percentage of dead adults (about 6.6%) has been shown already at the lowest percentage (6.25%). These data were statistically significant respecting to the control (*p* < 0.05) and others used concentrations (*p* < 0.0001; see also Supplementary Table S4). At 100%, about 30% of dead adults have been observed respect lower (0% and 12.5%; *p* < 0.0001 and *p* < 0.001, respectively) and higher (25% and 50%; *p* < 0.001) tested concentrations. On the basis of toxicity observed for T_1_, at 25%, 50%, and 100%, a significant decrease of survival (about 13%, 20%, and 23%, respectively) respecting lower concentrations (0%, 6.25%, and 12.5%; *p* < 0.0001 (Supplementary Table S4) was detected. Moreover, the data reported at 25% were statistically significant respecting to 50% (*p* < 0.05) and 100% (*p* < 0.001). Considering T_2_, T_3_, and T_4_ only at 50% and 100%, a significant decrease of survival (about 10%) respects all lower concentrations (*p* < 0.0001) and among them (*p* < 0.001; Supplementary Table S4).

W + PAHs condition at T_0_ and T_1_ caused a little survival decrease (about 10%) already at 6.25% that was statistically significant respecting to control (*p* < 0.0001) and other concentrations (*p* < 0.0001; Fig. 4B; Supplementary Table S4). At 25%, a significant decrease of about 50% respecting lower concentrations (*p* < 0.0001; Supplementary Table S4) and higher concentrations (50% and 100%; *p* < 0.0001) was detected. From T_2_ to T_6_, the results were similar. In fact, at 25%, a significant decrease of about 30% respecting lower concentrations (*p* < 0.0001 (Supplementary Table S4) and higher concentrations (50% and 100%; *p* < 0.0001) has been shown.

Considering W + PAHs + nZVI condition at T_0_ and T_1_ (Fig. 4C), a low percentage of about 25% of dead nauplii was observed at 12.5%. At 100%, significant increase of toxicity (about 80%) respecting lower (0%, 6.25%, and 12.5%; *p* < 0.0001) and higher (25% and 50%; *p* < 0.0001) tested concentrations was detected (Supplementary Table S4). Considering T_2_, T_3_, T_4_, and T_5_ only at 50% and 100%, a significant decrease of survival (about 10%) respects all lower concentrations (*p* < 0.0001) and among them (*p* < 0.0001; Supplementary Table S4). At T_6_, no toxicity was found.

Take into the consideration W + SED1 + nZVI condition at T_0_, T_1_, and T_3_ (Fig. 4D), a low percentage of dead nauplii (about 10%) was found at 50%. At 100%, significant increase of toxicity (about 20–30%) respect lower (0%, 6.25%, and 12.5%; p < 0.0001) and higher (25% and 50%; *p* < 0.0001) tested concentrations was observed (Supplementary Table S4). At T_4_, T_5_, and T_6_, no toxicity was displayed.

Only the 50% and 100% W + SED2 + nZVI solutions, collected from time 0 to time 4 (Fig. 4E) caused a significant decrease of survival (about 10% and 20%, respectively) respect all lower concentrations (*p* < 0.0001). At T_5_ and T_6_, no toxicity was found.

**Fig. 5:**
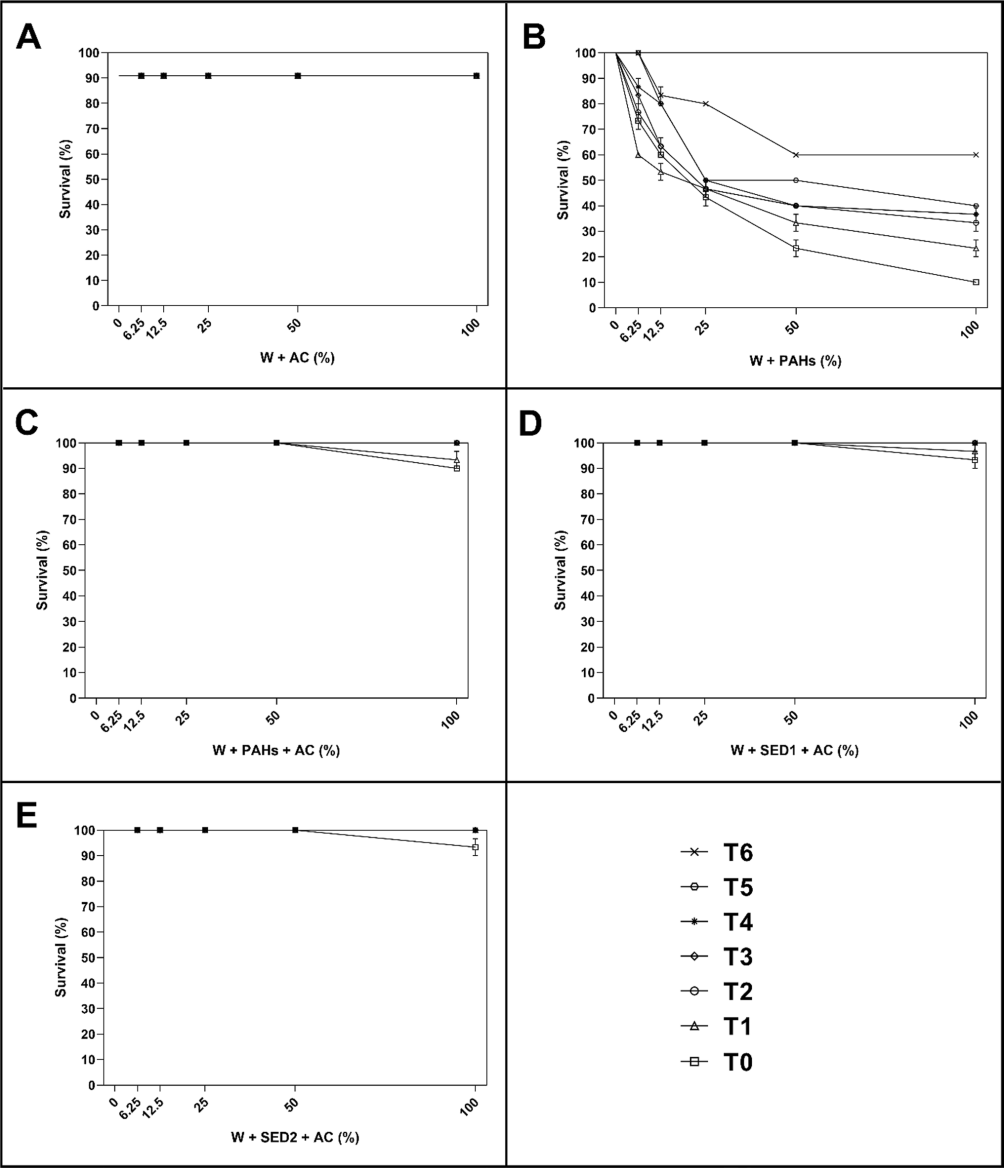
After 48 h, the percentage of surviving adults detected at seven time (T_0_ = after adding amendment; T_1_ = 3 h; T_2_ = 6 h; T_3_ = 21 h; T_4_ = 24 h; T_5_ = 72 h; and T_6_ = 21 days) both in control (0%) and treated samples with 6.25%, 12.5%, 25%, 50%, and 100% of **A** W + AC; **B** W + PAHs; **C** W + PAHs + AC; **D** W + SED1 + AC; and **E** W + SED1 + AC. Data are reported as mean ± standard deviation

As reported in Fig. 5, after 48 h of exposure to different percentage of aqueous solutions of all experimental conditions of AC, the scenario was a little different.

Considering W + AC condition (Fig. 5A), at all experimental times, no toxicity was showed. At T_0_ and T_1_, taking into consideration W + PAHs + AC (Fig. 5C), only at 100%, significant percentage of dead adults (about 10%) was measured respecting other concentrations (0%, 6.25%, 12.5%, 25%, and 50%; *p* < 0.0001, see also Supplementary Table S5). When considered the exposure to solutions collected at T_2_, T_3_, T_4_, T_5_, and T_6_, no dead organism was observed (Fig. 5C). At same manner, taking into consideration W + SED1 + AC (Fig. 5C), only at time 0 and 1, significant percentage of dead adults (about 10%) was showed, testing 100% of solutions respecting other concentrations (0%, 6.25%, 12.5%, 25%, and 50%; *p* < 0.0001 and *p* < 0.05 at T_0_ and T_1_, respectively; see also Supplementary Table S5).

Only when considered W + SED2 + AC (Fig. 5E), at 100% of T_0_ solutions, significant percentage of dead adults (from about 10%) was observed respecting other concentrations (*p* < 0.0001, see also Supplementary Table S5). At T_1_, T_2_, T_3_, T_4_, T_5_, and T_6_, no decrease of survival adults was observed.

### Effects of nZVI and AC on gene expression by real-time qPCR

**Fig. 6:**
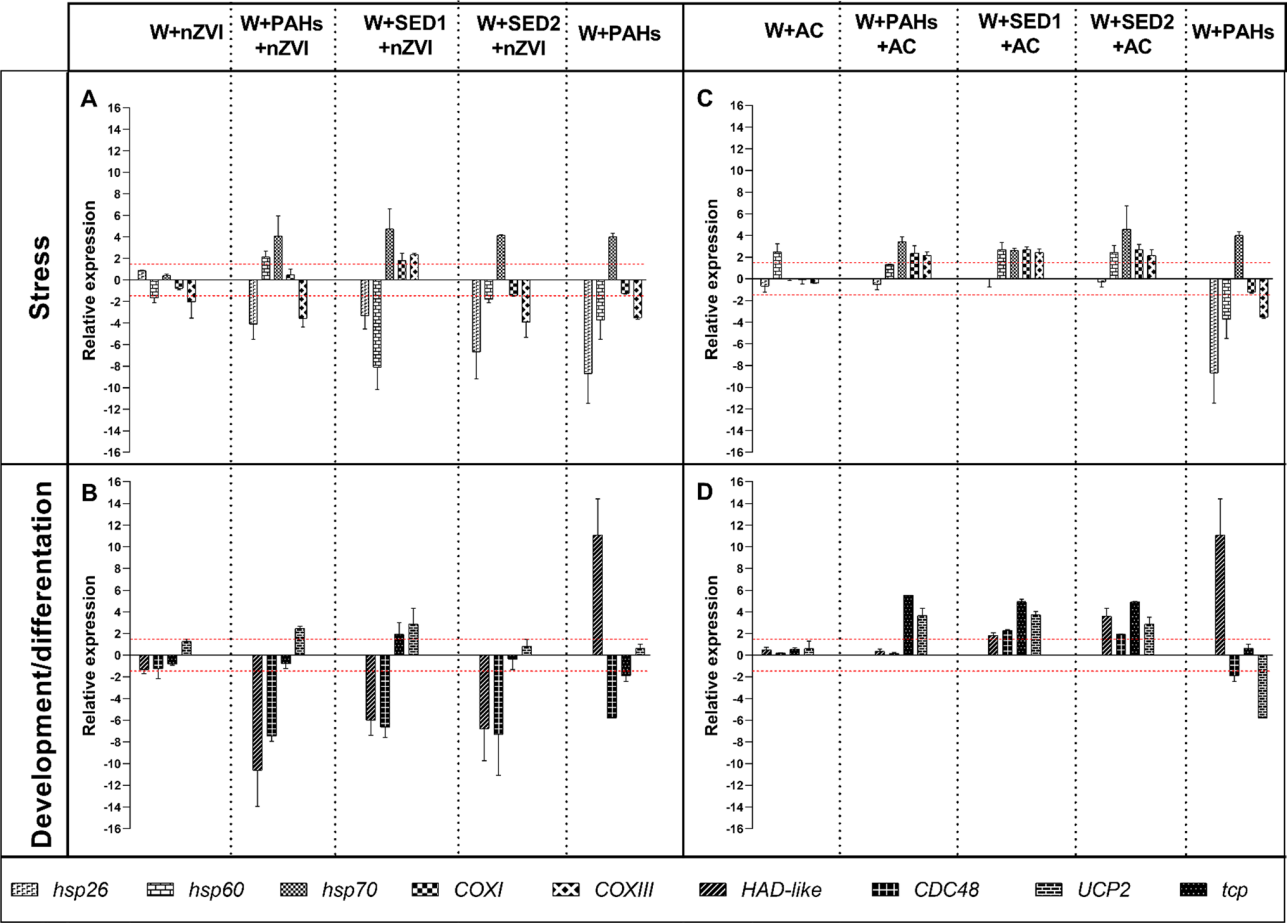
Histograms show the differences in expression levels of nine genes involved in stress response and in developmental processes. *A. franciscana* nauplii were exposed to W + nZVI, W + PAHs, W + PAHs + nZVI, W + SED1 + nZVI, W + SED2 + nZVI, W + AC, W + PAHs, W + PAHs + AC, W + SED1 + AC, and W + SED2 + AC at 100%. Expression levels of genes involved in stress response (**A**) and in developmental processes (**B**) after nZVI experiments; and expression levels of genes involved in stress response (**C**) and in developmental processes (**D**) after AC treatments. Fold differences greater than ± 1.5 (see red dotted horizontal guidelines at values of + 1.5 and − 1.5) were considered significant (see Supplementary Tables S6–7 for the values)

The expression levels of nine genes (Chen et al. 2009), involved in different physiological processes, were followed by real-time qPCR after nZVI remediation experiment (Fig. 6; see also Supplementary Table S6 for the values). Considering the stress response (Fig. 6A), *hsp 60* and *COXIII* were targeted in all experimental conditions. Specifically, *hsp 60* was downregulated by W + nZVI, W + PAHs, W + SED1 + nZVI, and W + SED2 + nZVI but was upregulated by W + PAHs + nZVI; *COXIII* was downregulated by W + nZVI, W + PAHs, W + PAHs + nZVI, and W + SED2 + nZVI but was upregulated by W + SED1 + nZVI. Moreover, *hsp 26* and *hsp70* were downregulated and upregulated, respectively, in all conditions with exception of W + nZVI. Finally, *COXI* was upregulated only by W + SED1 + nZVI (see Table S6).

Taking into the consideration the genes involved in developmental processes (Fig. 6B), four genes were targeted by all conditions with exception of W + nZVI. Common molecular targets for all conditions were *HAD*-like and *CDC48*, of which *HAD*-like was downregulated by W + PAHs + nZVI, W + SED1 + nZVI, and W + SED2 + nZVI and upregulated only by W + PAHs, whereas *CDC48* was downregulated by all conditions. The gene *tcp* was upregulated by W + SED1 + nZVI and downregulated by W + PAHs, and *UCP2* was upregulated by W + PAHs + nZVI and W + SED1 + nZVI (see Table S6). Also, after AC remediation experiment, these nine genes expression levels were evaluated (Fig. 6; see also Supplementary Table S7 for the values).

Evaluating the stress response, three *heat shock protein*s genes were targets for almost all test conditions. In particular, *hsp 60* was downregulated by W + PAHs and upregulated by W + AC, W + SED1 + AC, and W + SED2 + AC, whereas *hsp 70* was downregulated by W + SED1 + AC and upregulated by W + PAHs, W + PAHs + AC, and W + SED2 + AC. The gene *hsp26* was molecular target only for W + PAHs showing a downregulation. Moreover, *COXI* and *COXIII* were upregulated by W + PAHs + AC, W + SED1 + AC, and W + SED2 + AC, and only *COXIII* was downregulated by W + PAHs (Fig. 6C). Considering the impact on developmental processes (Fig. 6D), also in this case, four genes were targeted by all conditions with exception of W + AC. Common molecular target for all conditions was *tcp*, which showed an up-regulation after W + PAHs + AC, W + SED1 + AC, and W + SED2 + AC treatment, and downregulation after W + PAHs exposure. The gene *HAD*-like was upregulated by W + PAHs, W + SED1 + AC, and W + SED2 + AC, whereas *CDC48* was downregulated by W + PAHs and upregulated by W + SED1 + AC and W + SED2 + AC. Finally, *UCP2* was upregulated by W + PAHs + AC, W + SED1 + nZVI, and W + SED2 + AC (see Table S7).

**Fig. 7:**
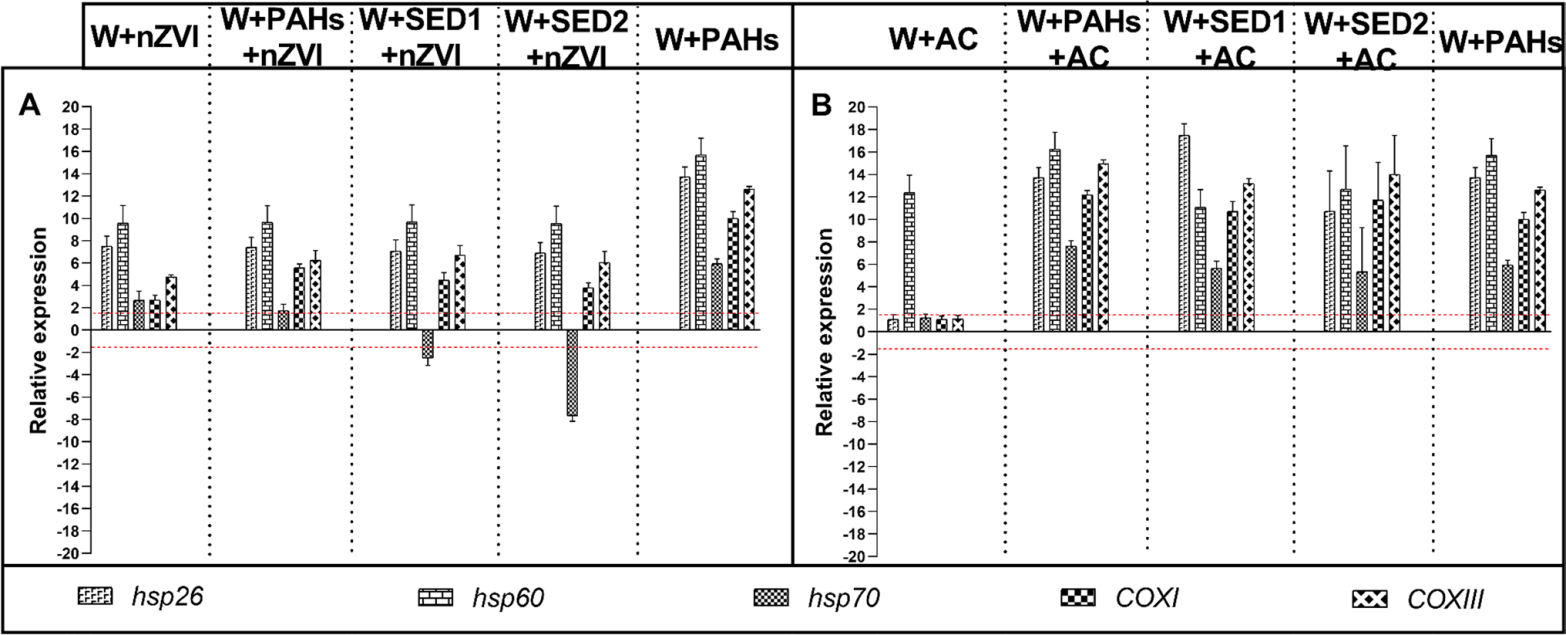
Histograms show the differences in expression levels of five genes involved in stress response. *A. franciscana* adults were exposed to W + nZVI, W + PAHs, W + PAHs + nZVI, W + SED1 + nZVI, and W + SED2 + nZVI, W + AC, W + PAHs, W + PAHs + AC, W + SED1 + AC, and W + SED2 + AC at 100%. Expression levels of genes involved in stress response after nZVI (A) and AC (B) experiments Fold differences greater than ± 1.5 (see red dotted horizontal guidelines at values of + 1.5 and − 1.5) were considered significant (see Supplementary Table S8–9 for the values)

As shown in Fig. 7, among the five genes analysed in adults of *A. franciscana*, all genes were targeted by all experimental conditions. Specifically, *hsp26*, *hsp60*, *COXI*, and *COXIII* were upregulated by all conditions of nZVI treatments (see Fig. 7A and Table S8), whereas *hsp70* was upregulated only by W + nZVI, W + PAHs, and W + PAHs + nZVI and downregulated by W + SED1 + nZVI and W + SED2 + nZVI (Supplementary Table S8). As shown Fig. 7B, all tested genes in adults were upregulated by all experimental conditions (W + AC, W + PAHs, W + PAHs + AC, W + SED1 + AC, and W + SED2 + AC treatments; Table S9).

## Discussion

Experiments of remediation with nZVI and AC were able to drastically decrease PAH concentration in aqueous solutions. In fact, nZVI and AC efficiency of removal were 99.6% and 98.3%, respectively. These results are in accordance to available literature. In fact, similar data showed after remediation experiment on aqueous solutions spiked both with trichloroethylene (TCE) and PAHs using a quantity of nZVI and AC corresponding to those used in the following study (Kim et al. 2010, 2014; Kumar et al. 2019).

When considered the removal of PAHs from sediment, we observed different results for the two sediments. In the case of sediment of Sarno (less polluted), both after AC and nZVI treatment, the PAHs were totally removal already after T_3_ (21 h). In fact, we observed the same percentage of removal, 98.9% (see Table 1). However, considering remediation of Bagnoli sediment (more polluted), nZVI and AC showed a percentage of removal of about 49.3% and 60.6%, respectively. Moreover, the results displayed that, for both nZVI and AC, the concentrations of the low molecular weight PAHs were reduced to a greater extent than that for the high molecular weight. In fact, as reported in Figs. S3–S6, these amendments was not able to completely remove the benzo[a]anthracene, indeno[1,2,3-cd]pyrene, and benzo[a]pyrene. Previous studies showed that for PAH-spiked sediments, activated carbon and nZVI addition significantly reduce the availability of low molecular weight products (Lebo et al. 2003; Chang et al. 2005; Zimmerman et al. 2005).

Significant mortality of *A. franciscana* nauplii and adults was observed upon exposure to aqueous solutions containing up to 3% of AC and nZVI (see Figs. 2, 3, 4, and 5). Specifically, considering the negative controls at T_0_, nZVI showed a little percentage of dead nauplii and adults (about 6.6%) already at the lowest percentage (6.25%), which was of about 30% at the maximum concentration (100%). Instead, AC displayed a small toxicity of about 10–20% only at the highest concentration. These results were in line with expectations shown in (Albarano et al. 2021a), where the AC was defined as safer (i.e., at low risk) than all other amendments on the basis of GHS criteria. Also, the data agree with previously reported observations of about 100% survival of different organisms, including *Daphnia magna*, on AC-enriched sediments (Cornelissen et al. 2006; Jonker et al. 2009; Lewis et al. 2016). This simple, first-tier response thus suggests the absence of harmful effects of AC addition. However, a closer look at the exposure systems revealed an indication for toxic effects of nZVI. Keller et al. (2012) and Jaafar and Yasid (2018) showed similar results (about 60% of mortality) exposing *D. magna* to nZVI for 28 days.

A result of particular interest was the genotoxicity on *A. franciscana*. The highest percentage of nauplii mortality caused by exposure to nZVI can be linked to the downregulation of the majority of the studied genes. Firstly, all 9 genes were molecular targets of this amendment, with the only exception of *hsp26*, *hsp70*, *COXI*, *HAD*-like, *CDC48*, *UCP2*, and *tcp*, which were not molecular target of negative control (Fig. 6A and B). However, the dangerous impact on adults by negative control can be linked to upregulation of all five genes involved in stress response (Fig. 7A). When considered the AC experiments, only *hsp60* was gene target of W + AC, emphasizing the low toxicity of this amendment (Figs. 6C, D and 7B).

Summarizing the real-time qPCR experiments of nZVI on nauplii, (i) two genes were targeted by all five experimental conditions, (ii) four genes were targeted by all experimental conditions with exception of negative control, (iii) one gene was only targets for W + PAHs and W + SED1 + nZVI, (iv) only one gene was specifically affected only by W + PAHs + nZVI and W + SED1 + nZVI, and (v) one gene was targeted by W + SED1 + nZVI. Considering the adults, (i) four genes was upregulated by all five experimental conditions; (ii) one gene was downregulated by W + SED1 + nZVI and W + SED2 + nZVI and upregulated by W + nZVI and W + PAHs + nZVI.

Taking into consideration experiment with AC on nauplii, we could observe the following: (i) only one gene was gene target of negative control; (ii) three genes were targeted by W + PAHs, W + SED1 + AC, and W + SED2 + AC; (iii) two genes were targeted by W + PAHs + AC, W + SED1 + AC, and W + SED2 + AC; (iv) two genes were targeted by W + PAHs, W + PAHs + AC, W + SED1 + AC, and W + SED2 + AC; (v) one gene was gene target only of W + PAHs. After adults’ exposure to all experimental conditions, (i) only one gene was gene target of negative control; (ii) five genes, involved in stress response, were targeted by all experimental conditions (W + AC, W + PAHs, W + PAHs + AC, W + SED1 + AC, and W + SED2 + AC).

The molecular response to nZVI appeared different in comparison with AC: negative control of nZVI (W + nZVI) downregulated two genes and all genes involved in stress response (see also Supplementary Materials, Supplementary Tables S6 and S8) in nauplii and in adults, respectively, compared to negative control of AC that upregulated only 1 gene (*hsp60*) both in nauplii and adults. In previous studies, nZVI genotoxicity was widely demonstrated. In fact, this amendment was able to cause high oxidative stress in plants *Allium* bulbs, in bacterial strains *Erwinia amylovora, Xantomonas oryzae, Bacillus cereus*, and *Streptomyces* spp., and also in *Artemia salina* (Barzan et al. 2014; Ghosh et al. 2017; Kumar et al. 2017).

All together, these molecular results revealed that the affected genes in *A. franciscana* were involved both in the stress response and development processes. In fact, all genes belonging to these classes were affected by all experimental conditions.

## Conclusions

Based on the results of this study, we have concluded that the use of nano-zero-valent iron (nZVI) and activated carbon (AC) is as follows: (i) effective methods for decreasing polycyclic aromatic hydrocarbons (PAHs) concentrations more in contaminated water (removal efficiency = 99%) than in polluted sediment, (ii) the survival of both nauplii and adults was mainly impacted by nZVI than by AC, and (iii) the nZVI- addition induced variations in the expression of genes, involved in stress response and developmental in both life stages. In conclusion, nZVI cannot be recommended as a remediating agent nevertheless its related efficiency due to its relative toxicity. Conversely, on a comparative basis, the AC can recommended despite its removal efficiency for most impacted sediment is not very high due to its reduced biological impact. Further research should stress on nZVI encapsulation, also with AC and natural-based polymers to strengthen its environmental compatibility on a one-health basis.

## Supplementary Information

Below is the link to the electronic supplementary material.

Supplementary file1 (DOCX 2105 KB)

## Data Availability

All data generated or analyzed during this study are included in this published article.
