# Supplementary material for: In situ microcosm remediation of polyaromatic hydrocarbons: influence and effectiveness of Nano-Zero Valent Iron and activated carbon
Source: Environ Sci Pollut Res Int. 2022 Aug 9;30(2):3235–51. doi: 10.1007/s11356-022-22408-y (PMC9892105; doi:10.1007/s11356-022-22408-y)

**Supplementary materials**

**Supplementary Table S1**. Initial concentrations of PAHs in sediments. The data were reported in µg/Kg.

|  | **SED 1** | **SED 2** |
| --- | --- | --- |
| **Acenaphthylene** | < 0.05 | 18.5 |
| **Anthracene** | 21.9 | 115.1 |
| **Phenanthrene** | < 0.05 | 58.4 |
| **Fluoranthene** | 23.6 | 323.0 |
| **Pyrene** | 22.5 | 282.9 |
| **Benzo(a)Antracene** | < 0.05 | 122.7 |
| **Chrysene** | 11.9 | 159.0 |
| **Benzo(b)Fluorantene** | < 0.05 | 201.6 |
| **Benzo[k]fluoranthene** | < 0.05 | 74.7 |
| **Benzo(e)Pirene** | < 0.05 | 119.4 |
| **Benzo[a]pyrene** | < 0.05 | 112.1 |
| **Indeno[1,2,3-cd]pyrene** | < 0.05 | 104.3 |
| **Benzo[ghi]perylene** | < 0.05 | 101.9 |
| **Total PAHs** | 80 | 1793.5 |

**Supplementary Table S2**. Tukey’s test used to indicate the significance level between nZVI different percentage and control on nauplii at all considered time (T_0_ = after adding of amendment; T_1_ = 3 h; T_2_ = 6 h; T_3_ = 21 h; T_4_ = 24 h, T_5_ = 72 h and T_6_ = 21 days): * < 0.05, ** p < 0.01, *** p < 0.001, **** p<0.0001. n.s. = not significant.

|  | **W + nZVI** | **W + PAHs** | **W + PAHs + nZVI** | **W + SED1 + nZVI** | **W + SED2 + nZVI** |
| --- | --- | --- | --- | --- | --- |
| **T_0_** |  |  |  |  |  |
| 0 vs. 6.25 | 0.0346 | 0.0001 | ns | ns | ns |
| 0 vs. 12.5 | <0.0001 | <0.0001 | <0.0001 | ns | ns |
| 0 vs. 25 | <0.0001 | <0.0001 | <0.0001 | ns | ns |
| 0 vs. 50 | <0.0001 | <0.0001 | <0.0001 | ns | <0.0001 |
| 0 vs. 100 | <0.0001 | <0.0001 | <0.0001 | <0.0001 | <0.0001 |
| 6.25 vs. 12.5 | <0.0001 | <0.0001 | <0.0001 | ns | ns |
| 6.25 vs. 25 | <0.0001 | <0.0001 | <0.0001 | ns | ns |
| 6.25 vs. 50 | <0.0001 | <0.0001 | <0.0001 | ns | <0.0001 |
| 6.25 vs. 100 | <0.0001 | <0.0001 | <0.0001 | <0.0001 | <0.0001 |
| 12.5 vs. 25 | ns | 0.0036 | ns | ns | ns |
| 12.5 vs. 50 | ns | <0.0001 | <0.0001 | ns | <0.0001 |
| 12.5 vs. 100 | 0.0002 | <0.0001 | <0.0001 | <0.0001 | <0.0001 |
| 25 vs. 50 | ns | ns | 0.0033 | ns | <0.0001 |
| 25 vs. 100 | 0.0002 | <0.0001 | <0.0001 | <0.0001 | <0.0001 |
| 50 vs. 100 | 0.0002 | <0.0001 | <0.0001 | <0.0001 | ns |
|  |  |  |  |  |  |
| **T_1_** |  |  |  |  |  |
| 0 vs. 6.25 | ns | ns | ns | ns | ns |
| 0 vs. 12.5 | ns | <0.0001 | ns | ns | ns |
| 0 vs. 25 | <0.0001 | <0.0001 | <0.0001 | ns | ns |
| 0 vs. 50 | <0.0001 | <0.0001 | <0.0001 | ns | <0.0001 |
| 0 vs. 100 | <0.0001 | <0.0001 | <0.0001 | <0.0001 | <0.0001 |
| 6.25 vs. 12.5 | ns | <0.0001 | ns | ns | ns |
| 6.25 vs. 25 | <0.0001 | <0.0001 | <0.0001 | ns | ns |
| 6.25 vs. 50 | <0.0001 | <0.0001 | <0.0001 | ns | <0.0001 |
| 6.25 vs. 100 | <0.0001 | <0.0001 | <0.0001 | <0.0001 | <0.0001 |
| 12.5 vs. 25 | <0.0001 | ns | <0.0001 | ns | ns |
| 12.5 vs. 50 | <0.0001 | <0.0001 | <0.0001 | ns | <0.0001 |
| 12.5 vs. 100 | <0.0001 | <0.0001 | <0.0001 | <0.0001 | <0.0001 |
| 25 vs. 50 | 0.0346 | <0.0001 | <0.0001 | ns | <0.0001 |
| 25 vs. 100 | 0.0002 | <0.0001 | <0.0001 | <0.0001 | <0.0001 |
| 50 vs. 100 | ns | <0.0001 | <0.0001 | <0.0001 | 0.0205 |
|  |  |  |  |  |  |
| **T_2_** |  |  |  |  |  |
| 0 vs. 6.25 | ns | ns | ns | ns | ns |
| 0 vs. 12.5 | ns | ns | ns | ns | ns |
| 0 vs. 25 | ns | <0.0001 | ns | ns | ns |
| 0 vs. 50 | <0.0001 | <0.0001 | 0.0033 | <0.0001 | <0.0001 |
| 0 vs. 100 | <0.0001 | <0.0001 | <0.0001 | <0.0001 | <0.0001 |
| 6.25 vs. 12.5 | ns | ns | ns | ns | ns |
| 6.25 vs. 25 | ns | <0.0001 | ns | ns | ns |
| 6.25 vs. 50 | <0.0001 | <0.0001 | 0.0033 | <0.0001 | <0.0001 |
| 6.25 vs. 100 | <0.0001 | <0.0001 | <0.0001 | <0.0001 | <0.0001 |
| 12.5 vs. 25 | ns | <0.0001 | ns | ns | ns |
| 12.5 vs. 50 | <0.0001 | <0.0001 | 0.0033 | <0.0001 | <0.0001 |
| 12.5 vs. 100 | <0.0001 | <0.0001 | <0.0001 | <0.0001 | <0.0001 |
| 25 vs. 50 | <0.0001 | ns | ns | <0.0001 | <0.0001 |
| 25 vs. 100 | <0.0001 | <0.0001 | <0.0001 | <0.0001 | <0.0001 |
| 50 vs. 100 | 0.0346 | <0.0001 | 0.0033 | <0.0001 | 0.0205 |
|  |  |  |  |  |  |
| **T_3_** |  |  |  |  |  |
| 0 vs. 6.25 | ns | ns | ns | ns | ns |
| 0 vs. 12.5 | ns | ns | ns | ns | ns |
| 0 vs. 25 | ns | <0.0001 | ns | ns | ns |
| 0 vs. 50 | 0.0346 | <0.0001 | ns | ns | <0.0001 |
| 0 vs. 100 | <0.0001 | <0.0001 | 0.0033 | <0.0001 | <0.0001 |
| 6.25 vs. 12.5 | ns | ns | ns | ns | ns |
| 6.25 vs. 25 | ns | <0.0001 | ns | ns | ns |
| 6.25 vs. 50 | 0.0346 | <0.0001 | ns | ns | <0.0001 |
| 6.25 vs. 100 | <0.0001 | <0.0001 | 0.0033 | <0.0001 | <0.0001 |
| 12.5 vs. 25 | ns | <0.0001 | ns | ns | ns |
| 12.5 vs. 50 | 0.0346 | <0.0001 | ns | ns | <0.0001 |
| 12.5 vs. 100 | <0.0001 | <0.0001 | 0.0033 | <0.0001 | <0.0001 |
| 25 vs. 50 | 0.0346 | ns | ns | ns | <0.0001 |
| 25 vs. 100 | <0.0001 | <0.0001 | 0.0033 | <0.0001 | <0.0001 |
| 50 vs. 100 | 0.0002 | 0.0036 | ns | <0.0001 | ns |
|  |  |  |  |  |  |
| **T_4_** |  |  |  |  |  |
| 0 vs. 6.25 | ns | ns | ns | ns | ns |
| 0 vs. 12.5 | ns | ns | ns | ns | ns |
| 0 vs. 25 | ns | <0.0001 | ns | ns | ns |
| 0 vs. 50 | ns | <0.0001 | ns | ns | 0.0205 |
| 0 vs. 100 | <0.0001 | <0.0001 | 0.0033 | 0.0005 | <0.0001 |
| 6.25 vs. 12.5 | ns | ns | ns | ns | ns |
| 6.25 vs. 25 | ns | <0.0001 | ns | ns | ns |
| 6.25 vs. 50 | ns | <0.0001 | ns | ns | 0.0205 |
| 6.25 vs. 100 | <0.0001 | <0.0001 | 0.0033 | 0.0005 | <0.0001 |
| 12.5 vs. 25 | ns | <0.0001 | ns | ns | ns |
| 12.5 vs. 50 | ns | <0.0001 | ns | ns | 0.0205 |
| 12.5 vs. 100 | <0.0001 | <0.0001 | 0.0033 | 0.0005 | <0.0001 |
| 25 vs. 50 | ns | ns | ns | ns | 0.0205 |
| 25 vs. 100 | <0.0001 | 0.0001 | 0.0033 | 0.0005 | <0.0001 |
| 50 vs. 100 | <0.0001 | 0.0036 | 0.0569 | 0.0005 | ns |
|  |  |  |  |  |  |
| **T_5_** |  |  |  |  |  |
| 0 vs. 6.25 | ns | ns | ns | ns | ns |
| 0 vs. 12.5 | ns | ns | ns | ns | ns |
| 0 vs. 25 | ns | ns | ns | ns | ns |
| 0 vs. 50 | ns | <0.0001 | ns | ns | ns |
| 0 vs. 100 | <0.0001 | <0.0001 | ns | ns | ns |
| 6.25 vs. 12.5 | ns | ns | ns | ns | ns |
| 6.25 vs. 25 | ns | <0.0001 | ns | ns | ns |
| 6.25 vs. 50 | ns | <0.0001 | ns | ns | ns |
| 6.25 vs. 100 | <0.0001 | <0.0001 | ns | ns | ns |
| 12.5 vs. 25 | ns | <0.0001 | ns | ns | ns |
| 12.5 vs. 50 | ns | <0.0001 | ns | ns | ns |
| 12.5 vs. 100 | <0.0001 | <0.0001 | ns | ns | ns |
| 25 vs. 50 | ns | ns | ns | ns | ns |
| 25 vs. 100 | <0.0001 | 0.0001 | ns | ns | ns |
| 50 vs. 100 | <0.0001 | 0.0036 | ns | ns | ns |
|  |  |  |  |  |  |
| **T_6_** |  |  |  |  |  |
| 0 vs. 6.25 | ns | ns | ns | ns | ns |
| 0 vs. 12.5 | ns | ns | ns | ns | ns |
| 0 vs. 25 | ns | <0.0001 | ns | ns | ns |
| 0 vs. 50 | ns | <0.0001 | ns | ns | ns |
| 0 vs. 100 | ns | <0.0001 | ns | ns | ns |
| 6.25 vs. 12.5 | ns | ns | ns | ns | ns |
| 6.25 vs. 25 | ns | <0.0001 | ns | ns | ns |
| 6.25 vs. 50 | ns | <0.0001 | ns | ns | ns |
| 6.25 vs. 100 | ns | <0.0001 | ns | ns | ns |
| 12.5 vs. 25 | ns | <0.0001 | ns | ns | ns |
| 12.5 vs. 50 | ns | <0.0001 | ns | ns | ns |
| 12.5 vs. 100 | ns | <0.0001 | ns | ns | ns |
| 25 vs. 50 | ns | ns | ns | ns | ns |
| 25 vs. 100 | ns | 0.0001 | ns | ns | ns |
| 50 vs. 100 | ns | 0.016 | ns | ns | ns |

**Supplementary Table S3**. Tukey’s test used to indicate the significance level between AC different percentage and control on naupli at all considered time (T_0_ = after adding of amendment; T_1_ = 3 h; T_2_ = 6 h; T_3_ = 21 h; T_4_ = 24 h, T_5_ = 72 h and T_6_ = 21 days): * < 0.05, ** p < 0.01, *** p < 0.001, **** p<0.0001. n.s. = not significant.

|  | **W + AC** | **W + PAHs** | **W + PAHs + AC** | **W + SED1 + AC** | **W + SED2 + AC** |
| --- | --- | --- | --- | --- | --- |
| **T_0_** |  |  |  |  |  |
| 0 vs. 6.25 | ns | <0.0001 | ns | ns | ns |
| 0 vs. 12.5 | ns | <0.0001 | ns | ns | ns |
| 0 vs. 25 | ns | <0.0001 | ns | ns | ns |
| 0 vs. 50 | ns | <0.0001 | ns | ns | ns |
| 0 vs. 100 | <0.0001 | <0.0001 | <0.0001 | <0.0001 | <0.0001 |
| 6.25 vs. 12.5 | ns | 0.0004 | ns | ns | ns |
| 6.25 vs. 25 | ns | <0.0001 | ns | ns | ns |
| 6.25 vs. 50 | ns | <0.0001 | ns | ns | ns |
| 6.25 vs. 100 | <0.0001 | <0.0001 | <0.0001 | <0.0001 | <0.0001 |
| 12.5 vs. 25 | ns | <0.0001 | ns | ns | ns |
| 12.5 vs. 50 | ns | <0.0001 | ns | ns | ns |
| 12.5 vs. 100 | <0.0001 | <0.0001 | <0.0001 | <0.0001 | <0.0001 |
| 25 vs. 50 | ns | <0.0001 | ns | ns | ns |
| 25 vs. 100 | <0.0001 | <0.0001 | <0.0001 | <0.0001 | <0.0001 |
| 50 vs. 100 | <0.0001 | 0.0004 | <0.0001 | <0.0001 | <0.0001 |
|  |  |  |  |  |  |
| **T_1_** |  |  |  |  |  |
| 0 vs. 6.25 | ns | <0.0001 | ns | ns | ns |
| 0 vs. 12.5 | ns | <0.0001 | ns | ns | ns |
| 0 vs. 25 | ns | <0.0001 | ns | ns | ns |
| 0 vs. 50 | ns | <0.0001 | ns | ns | ns |
| 0 vs. 100 | 0.0205 | <0.0001 | <0.0001 | <0.0001 | <0.0001 |
| 6.25 vs. 12.5 | ns | 0.2384 | ns | ns | ns |
| 6.25 vs. 25 | ns | 0.0004 | ns | ns | ns |
| 6.25 vs. 50 | ns | <0.0001 | ns | ns | ns |
| 6.25 vs. 100 | 0.0205 | <0.0001 | <0.0001 | <0.0001 | <0.0001 |
| 12.5 vs. 25 | ns | 0.2384 | ns | ns | ns |
| 12.5 vs. 50 | ns | <0.0001 | ns | ns | ns |
| 12.5 vs. 100 | 0.0205 | <0.0001 | <0.0001 | <0.0001 | <0.0001 |
| 25 vs. 50 | ns | 0.0004 | ns | ns | ns |
| 25 vs. 100 | 0.0205 | <0.0001 | <0.0001 | <0.0001 | <0.0001 |
| 50 vs. 100 | 0.0205 | 0.0156 | <0.0001 | <0.0001 | <0.0001 |
|  |  |  |  |  |  |
| **T_2_** |  |  |  |  |  |
| 0 vs. 6.25 | ns | <0.0001 | ns | ns | ns |
| 0 vs. 12.5 | ns | <0.0001 | ns | ns | ns |
| 0 vs. 25 | ns | <0.0001 | ns | ns | ns |
| 0 vs. 50 | ns | <0.0001 | ns | ns | ns |
| 0 vs. 100 | ns | <0.0001 | <0.0001 | ns | <0.0001 |
| 6.25 vs. 12.5 | ns | 0.0004 | ns | ns | ns |
| 6.25 vs. 25 | ns | <0.0001 | ns | ns | ns |
| 6.25 vs. 50 | ns | <0.0001 | ns | ns | ns |
| 6.25 vs. 100 | ns | <0.0001 | <0.0001 | ns | <0.0001 |
| 12.5 vs. 25 | ns | <0.0001 | ns | ns | ns |
| 12.5 vs. 50 | ns | <0.0001 | ns | ns | ns |
| 12.5 vs. 100 | ns | <0.0001 | <0.0001 | ns | <0.0001 |
| 25 vs. 50 | ns | ns | ns | ns | ns |
| 25 vs. 100 | ns | 0.0004 | <0.0001 | ns | <0.0001 |
| 50 vs. 100 | ns | ns | <0.0001 | ns | <0.0001 |
|  |  |  |  |  |  |
| **T_3_** |  |  |  |  |  |
| 0 vs. 6.25 | ns | <0.0001 | ns | ns | ns |
| 0 vs. 12.5 | ns | <0.0001 | ns | ns | ns |
| 0 vs. 25 | ns | <0.0001 | ns | ns | ns |
| 0 vs. 50 | ns | <0.0001 | ns | ns | ns |
| 0 vs. 100 | ns | <0.0001 | ns | ns | <0.0001 |
| 6.25 vs. 12.5 | ns | <0.0001 | ns | ns | ns |
| 6.25 vs. 25 | ns | <0.0001 | ns | ns | ns |
| 6.25 vs. 50 | ns | <0.0001 | ns | ns | ns |
| 6.25 vs. 100 | ns | <0.0001 | ns | ns | <0.0001 |
| 12.5 vs. 25 | ns | <0.0001 | ns | ns | ns |
| 12.5 vs. 50 | ns | <0.0001 | ns | ns | ns |
| 12.5 vs. 100 | ns | <0.0001 | ns | ns | <0.0001 |
| 25 vs. 50 | ns | ns | ns | ns | ns |
| 25 vs. 100 | ns | 0.0156 | ns | ns | <0.0001 |
| 50 vs. 100 | ns | ns | ns | ns | <0.0001 |
|  |  |  |  |  |  |
| **T_4_** |  |  |  |  |  |
| 0 vs. 6.25 | ns | 0.0004 | ns | ns | ns |
| 0 vs. 12.5 | ns | <0.0001 | ns | ns | ns |
| 0 vs. 25 | ns | <0.0001 | ns | ns | ns |
| 0 vs. 50 | ns | <0.0001 | ns | ns | ns |
| 0 vs. 100 | ns | <0.0001 | ns | ns | ns |
| 6.25 vs. 12.5 | ns | ns | ns | ns | ns |
| 6.25 vs. 25 | ns | <0.0001 | ns | ns | ns |
| 6.25 vs. 50 | ns | <0.0001 | ns | ns | ns |
| 6.25 vs. 100 | ns | <0.0001 | ns | ns | ns |
| 12.5 vs. 25 | ns | <0.0001 | ns | ns | ns |
| 12.5 vs. 50 | ns | <0.0001 | ns | ns | ns |
| 12.5 vs. 100 | ns | <0.0001 | ns | ns | ns |
| 25 vs. 50 | ns | 0.0156 | ns | ns | ns |
| 25 vs. 100 | ns | 0.0004 | ns | ns | ns |
| 50 vs. 100 | ns | ns | ns | ns | ns |
|  |  |  |  |  |  |
| **T_5_** |  |  |  |  |  |
| 0 vs. 6.25 | ns | ns | ns | ns | ns |
| 0 vs. 12.5 | ns | <0.0001 | ns | ns | ns |
| 0 vs. 25 | ns | <0.0001 | ns | ns | ns |
| 0 vs. 50 | ns | <0.0001 | ns | ns | ns |
| 0 vs. 100 | ns | <0.0001 | ns | ns | ns |
| 6.25 vs. 12.5 | ns | <0.0001 | ns | ns | ns |
| 6.25 vs. 25 | ns | <0.0001 | ns | ns | ns |
| 6.25 vs. 50 | ns | <0.0001 | ns | ns | ns |
| 6.25 vs. 100 | ns | <0.0001 | ns | ns | ns |
| 12.5 vs. 25 | ns | <0.0001 | ns | ns | ns |
| 12.5 vs. 50 | ns | <0.0001 | ns | ns | ns |
| 12.5 vs. 100 | ns | <0.0001 | ns | ns | ns |
| 25 vs. 50 | ns | ns | ns | ns | ns |
| 25 vs. 100 | ns | 0.0156 | ns | ns | ns |
| 50 vs. 100 | ns | 0.0156 | ns | ns | ns |
|  |  |  |  |  |  |
| **T_6_** |  |  |  |  |  |
| 0 vs. 6.25 | ns | ns | ns | ns | ns |
| 0 vs. 12.5 | ns | <0.0001 | ns | ns | ns |
| 0 vs. 25 | ns | <0.0001 | ns | ns | ns |
| 0 vs. 50 | ns | <0.0001 | ns | ns | ns |
| 0 vs. 100 | ns | <0.0001 | ns | ns | ns |
| 6.25 vs. 12.5 | ns | <0.0001 | ns | ns | ns |
| 6.25 vs. 25 | ns | <0.0001 | ns | ns | ns |
| 6.25 vs. 50 | ns | <0.0001 | ns | ns | ns |
| 6.25 vs. 100 | ns | <0.0001 | ns | ns | ns |
| 12.5 vs. 25 | ns | <0.0001 | ns | ns | ns |
| 12.5 vs. 50 | ns | <0.0001 | ns | ns | ns |
| 12.5 vs. 100 | ns | <0.0001 | ns | ns | ns |
| 25 vs. 50 | ns | ns | ns | ns | ns |
| 25 vs. 100 | ns | 0.0156 | ns | ns | ns |
| 50 vs. 100 | ns | 0.0156 | ns | ns | ns |

**Supplementary Table S4**. Tukey’s test used to indicate the significance level between nZVI different percentage and control on adults at all considered time (T_0_ = after adding of amendment; T_1_ = 3 h; T_2_ = 6 h; T_3_ = 21 h; T4 = 24 h, T_5_ = 72 h and T_6_ = 21 days): * < 0.05, ** p < 0.01, *** p < 0.001, **** p<0.0001. n.s. = not significant.

|  | **W + nZVI** | **W + PAHs** | **W + PAHs + nZVI** | **W + SED1 + nZVI** | **W + SED2 + nZVI** |
| --- | --- | --- | --- | --- | --- |
| **T_0_** |  |  |  |  |  |
| 0 vs. 6.25 | 0.0346 | <0.0001 | ns | ns | ns |
| 0 vs. 12.5 | <0.0001 | <0.0001 | <0.0001 | ns | ns |
| 0 vs. 25 | <0.0001 | <0.0001 | <0.0001 | ns | ns |
| 0 vs. 50 | <0.0001 | <0.0001 | <0.0001 | ns | <0.0001 |
| 0 vs. 100 | <0.0001 | <0.0001 | <0.0001 | <0.0001 | <0.0001 |
| 6.25 vs. 12.5 | <0.0001 | 0.0006 | <0.0001 | ns | ns |
| 6.25 vs. 25 | <0.0001 | <0.0001 | <0.0001 | ns | ns |
| 6.25 vs. 50 | <0.0001 | <0.0001 | <0.0001 | ns | <0.0001 |
| 6.25 vs. 100 | <0.0001 | <0.0001 | <0.0001 | <0.0001 | <0.0001 |
| 12.5 vs. 25 | ns | <0.0001 | 0.002 | ns | ns |
| 12.5 vs. 50 | ns | <0.0001 | <0.0001 | ns | <0.0001 |
| 12.5 vs. 100 | 0.0002 | <0.0001 | <0.0001 | <0.0001 | <0.0001 |
| 25 vs. 50 | ns | <0.0001 | <0.0001 | ns | <0.0001 |
| 25 vs. 100 | 0.0002 | <0.0001 | <0.0001 | <0.0001 | <0.0001 |
| 50 vs. 100 | 0.0002 | 0.0006 | <0.0001 | <0.0001 | 0.0002 |
|  |  |  |  |  |  |
| **T_1_** |  |  |  |  |  |
| 0 vs. 6.25 | ns | <0.0001 | ns | ns | ns |
| 0 vs. 12.5 | ns | <0.0001 | ns | ns | <0.0001 |
| 0 vs. 25 | <0.0001 | <0.0001 | <0.0001 | ns | <0.0001 |
| 0 vs. 50 | <0.0001 | <0.0001 | <0.0001 | ns | <0.0001 |
| 0 vs. 100 | <0.0001 | <0.0001 | <0.0001 | <0.0001 | <0.0001 |
| 6.25 vs. 12.5 | ns | ns | ns | ns | <0.0001 |
| 6.25 vs. 25 | <0.0001 | 0.0006 | <0.0001 | ns | <0.0001 |
| 6.25 vs. 50 | <0.0001 | <0.0001 | <0.0001 | ns | <0.0001 |
| 6.25 vs. 100 | <0.0001 | <0.0001 | <0.0001 | <0.0001 | <0.0001 |
| 12.5 vs. 25 | <0.0001 | ns | <0.0001 | ns | <0.0001 |
| 12.5 vs. 50 | <0.0001 | <0.0001 | <0.0001 | ns | <0.0001 |
| 12.5 vs. 100 | <0.0001 | <0.0001 | <0.0001 | <0.0001 | <0.0001 |
| 25 vs. 50 | 0.0346 | 0.0006 | <0.0001 | ns | ns |
| 25 vs. 100 | 0.0002 | <0.0001 | <0.0001 | <0.0001 | ns |
| 50 vs. 100 | ns | 0.0205 | <0.0001 | <0.0001 | ns |
|  |  |  |  |  |  |
| **T_2_** |  |  |  |  |  |
| 0 vs. 6.25 | ns | <0.0001 | ns | ns | ns |
| 0 vs. 12.5 | ns | <0.0001 | <0.0001 | ns | ns |
| 0 vs. 25 | ns | <0.0001 | <0.0001 | ns | <0.0001 |
| 0 vs. 50 | <0.0001 | <0.0001 | <0.0001 | <0.0001 | <0.0001 |
| 0 vs. 100 | <0.0001 | <0.0001 | <0.0001 | <0.0001 | <0.0001 |
| 6.25 vs. 12.5 | ns | 0.0006 | <0.0001 | ns | ns |
| 6.25 vs. 25 | ns | <0.0001 | <0.0001 | ns | <0.0001 |
| 6.25 vs. 50 | <0.0001 | <0.0001 | <0.0001 | <0.0001 | <0.0001 |
| 6.25 vs. 100 | <0.0001 | <0.0001 | <0.0001 | <0.0001 | <0.0001 |
| 12.5 vs. 25 | ns | <0.0001 | ns | ns | <0.0001 |
| 12.5 vs. 50 | <0.0001 | <0.0001 | <0.0001 | <0.0001 | <0.0001 |
| 12.5 vs. 100 | <0.0001 | <0.0001 | <0.0001 | <0.0001 | <0.0001 |
| 25 vs. 50 | <0.0001 | ns | <0.0001 | <0.0001 | ns |
| 25 vs. 100 | <0.0001 | 0.0006 | <0.0001 | <0.0001 | ns |
| 50 vs. 100 | 0.0346 | ns | ns | <0.0001 | ns |
|  |  |  |  |  |  |
| **T_3_** |  |  |  |  |  |
| 0 vs. 6.25 | ns | <0.0001 | ns | ns | ns |
| 0 vs. 12.5 | ns | <0.0001 | <0.0001 | ns | ns |
| 0 vs. 25 | ns | <0.0001 | <0.0001 | ns | <0.0001 |
| 0 vs. 50 | 0.0346 | <0.0001 | <0.0001 | ns | <0.0001 |
| 0 vs. 100 | <0.0001 | <0.0001 | <0.0001 | <0.0001 | <0.0001 |
| 6.25 vs. 12.5 | ns | <0.0001 | <0.0001 | ns | ns |
| 6.25 vs. 25 | ns | <0.0001 | <0.0001 | ns | <0.0001 |
| 6.25 vs. 50 | 0.0346 | <0.0001 | <0.0001 | ns | <0.0001 |
| 6.25 vs. 100 | <0.0001 | <0.0001 | <0.0001 | <0.0001 | <0.0001 |
| 12.5 vs. 25 | ns | <0.0001 | ns | ns | <0.0001 |
| 12.5 vs. 50 | 0.0346 | <0.0001 | <0.0001 | ns | <0.0001 |
| 12.5 vs. 100 | <0.0001 | <0.0001 | <0.0001 | <0.0001 | <0.0001 |
| 25 vs. 50 | 0.0346 | ns | <0.0001 | ns | ns |
| 25 vs. 100 | <0.0001 | 0.0205 | <0.0001 | <0.0001 | ns |
| 50 vs. 100 | 0.0002 | ns | ns | <0.0001 | ns |
|  |  |  |  |  |  |
| **T_4_** |  |  |  |  |  |
| 0 vs. 6.25 | ns | 0.0006 | ns | ns | ns |
| 0 vs. 12.5 | ns | <0.0001 | <0.0001 | ns | ns |
| 0 vs. 25 | ns | <0.0001 | <0.0001 | ns | <0.0001 |
| 0 vs. 50 | ns | <0.0001 | <0.0001 | ns | <0.0001 |
| 0 vs. 100 | <0.0001 | <0.0001 | <0.0001 | 0.0005 | <0.0001 |
| 6.25 vs. 12.5 | ns | ns | <0.0001 | ns | ns |
| 6.25 vs. 25 | ns | <0.0001 | <0.0001 | ns | <0.0001 |
| 6.25 vs. 50 | ns | <0.0001 | <0.0001 | ns | <0.0001 |
| 6.25 vs. 100 | <0.0001 | <0.0001 | <0.0001 | 0.0005 | <0.0001 |
| 12.5 vs. 25 | ns | <0.0001 | ns | ns | <0.0001 |
| 12.5 vs. 50 | ns | <0.0001 | <0.0001 | ns | <0.0001 |
| 12.5 vs. 100 | <0.0001 | <0.0001 | <0.0001 | 0.0005 | <0.0001 |
| 25 vs. 50 | ns | 0.0205 | <0.0001 | ns | ns |
| 25 vs. 100 | <0.0001 | 0.0006 | <0.0001 | 0.0005 | ns |
| 50 vs. 100 | <0.0001 | ns | ns | 0.0005 | ns |
|  |  |  |  |  |  |
| **T_5_** |  |  |  |  |  |
| 0 vs. 6.25 | ns | ns | ns | ns | ns |
| 0 vs. 12.5 | ns | <0.0001 | <0.0001 | ns | ns |
| 0 vs. 25 | ns | <0.0001 | <0.0001 | ns | <0.0001 |
| 0 vs. 50 | ns | <0.0001 | <0.0001 | ns | <0.0001 |
| 0 vs. 100 | <0.0001 | <0.0001 | <0.0001 | ns | <0.0001 |
| 6.25 vs. 12.5 | ns | <0.0001 | <0.0001 | ns | ns |
| 6.25 vs. 25 | ns | <0.0001 | <0.0001 | ns | <0.0001 |
| 6.25 vs. 50 | ns | <0.0001 | <0.0001 | ns | <0.0001 |
| 6.25 vs. 100 | <0.0001 | <0.0001 | <0.0001 | ns | <0.0001 |
| 12.5 vs. 25 | ns | <0.0001 | ns | ns | <0.0001 |
| 12.5 vs. 50 | ns | <0.0001 | <0.0001 | ns | <0.0001 |
| 12.5 vs. 100 | <0.0001 | <0.0001 | <0.0001 | ns | <0.0001 |
| 25 vs. 50 | ns | ns | <0.0001 | ns | ns |
| 25 vs. 100 | <0.0001 | 0.0205 | <0.0001 | ns | ns |
| 50 vs. 100 | <0.0001 | 0.0205 | ns | ns | ns |
|  |  |  |  |  |  |
| **T_6_** |  |  |  |  |  |
| 0 vs. 6.25 | ns | ns | ns | ns | ns |
| 0 vs. 12.5 | ns | <0.0001 | <0.0001 | ns | ns |
| 0 vs. 25 | ns | <0.0001 | <0.0001 | ns | <0.0001 |
| 0 vs. 50 | ns | <0.0001 | <0.0001 | ns | <0.0001 |
| 0 vs. 100 | ns | <0.0001 | <0.0001 | ns | <0.0001 |
| 6.25 vs. 12.5 | ns | <0.0001 | <0.0001 | ns | ns |
| 6.25 vs. 25 | ns | <0.0001 | <0.0001 | ns | <0.0001 |
| 6.25 vs. 50 | ns | <0.0001 | <0.0001 | ns | <0.0001 |
| 6.25 vs. 100 | ns | <0.0001 | <0.0001 | ns | <0.0001 |
| 12.5 vs. 25 | ns | ns | ns | ns | <0.0001 |
| 12.5 vs. 50 | ns | <0.0001 | <0.0001 | ns | <0.0001 |
| 12.5 vs. 100 | ns | <0.0001 | <0.0001 | ns | <0.0001 |
| 25 vs. 50 | ns | <0.0001 | <0.0001 | ns | ns |
| 25 vs. 100 | ns | <0.0001 | <0.0001 | ns | ns |
| 50 vs. 100 | ns | ns | ns | ns | ns |

**Supplementary Table S5**. Tukey’s test used to indicate the significance level between AC different percentage and control on adults at all considered time (T_0_ = after adding of amendment; T_1_ = 3 h; T_2_ = 6 h; T_3_ = 21 h; T_4_ = 24 h, T_5_ = 72 h and T_6_ = 21 days): * < 0.05, ** p < 0.01, *** p < 0.001, **** p<0.0001. n.s. = not significant.

|  | **W + AC** | **W + PAHs** | **W + PAHs + AC** | **W + SED1 + AC** | **W + SED2 + AC** |
| --- | --- | --- | --- | --- | --- |
| **T_0_** |  |  |  |  |  |
| 0 vs. 6.25 | ns | <0.0001 | ns | ns | ns |
| 0 vs. 12.5 | ns | <0.0001 | ns | ns | ns |
| 0 vs. 25 | ns | <0.0001 | ns | ns | ns |
| 0 vs. 50 | ns | <0.0001 | ns | ns | ns |
| 0 vs. 100 | ns | <0.0001 | <0.0001 | <0.0001 | <0.0001 |
| 6.25 vs. 12.5 | ns | 0.0004 | ns | ns | ns |
| 6.25 vs. 25 | ns | <0.0001 | ns | ns | ns |
| 6.25 vs. 50 | ns | <0.0001 | ns | ns | ns |
| 6.25 vs. 100 | ns | <0.0001 | <0.0001 | <0.0001 | <0.0001 |
| 12.5 vs. 25 | ns | <0.0001 | ns | ns | ns |
| 12.5 vs. 50 | ns | <0.0001 | ns | ns | ns |
| 12.5 vs. 100 | ns | <0.0001 | <0.0001 | <0.0001 | <0.0001 |
| 25 vs. 50 | ns | <0.0001 | ns | ns | ns |
| 25 vs. 100 | ns | <0.0001 | <0.0001 | <0.0001 | <0.0001 |
| 50 vs. 100 | ns | 0.0004 | <0.0001 | <0.0001 | <0.0001 |
|  |  |  |  |  |  |
| **T_1_** |  |  |  |  |  |
| 0 vs. 6.25 | ns | <0.0001 | ns | ns | ns |
| 0 vs. 12.5 | ns | <0.0001 | ns | ns | ns |
| 0 vs. 25 | ns | <0.0001 | ns | ns | ns |
| 0 vs. 50 | ns | <0.0001 | ns | ns | ns |
| 0 vs. 100 | ns | <0.0001 | <0.0001 | 0.0205 | ns |
| 6.25 vs. 12.5 | ns | 0.2384 | ns | ns | ns |
| 6.25 vs. 25 | ns | 0.0004 | ns | ns | ns |
| 6.25 vs. 50 | ns | <0.0001 | ns | ns | ns |
| 6.25 vs. 100 | ns | <0.0001 | <0.0001 | 0.0205 | ns |
| 12.5 vs. 25 | ns | 0.2384 | ns | ns | ns |
| 12.5 vs. 50 | ns | <0.0001 | ns | ns | ns |
| 12.5 vs. 100 | ns | <0.0001 | <0.0001 | 0.0205 | ns |
| 25 vs. 50 | ns | 0.0004 | ns | ns | ns |
| 25 vs. 100 | ns | <0.0001 | <0.0001 | 0.0205 | ns |
| 50 vs. 100 | ns | 0.0156 | <0.0001 | 0.0205 | ns |
|  |  |  |  |  |  |
| **T_2_** |  |  |  |  |  |
| 0 vs. 6.25 | ns | <0.0001 | ns | ns | ns |
| 0 vs. 12.5 | ns | <0.0001 | ns | ns | ns |
| 0 vs. 25 | ns | <0.0001 | ns | ns | ns |
| 0 vs. 50 | ns | <0.0001 | ns | ns | ns |
| 0 vs. 100 | ns | <0.0001 | ns | ns | ns |
| 6.25 vs. 12.5 | ns | 0.0004 | ns | ns | ns |
| 6.25 vs. 25 | ns | <0.0001 | ns | ns | ns |
| 6.25 vs. 50 | ns | <0.0001 | ns | ns | ns |
| 6.25 vs. 100 | ns | <0.0001 | ns | ns | ns |
| 12.5 vs. 25 | ns | <0.0001 | ns | ns | ns |
| 12.5 vs. 50 | ns | <0.0001 | ns | ns | ns |
| 12.5 vs. 100 | ns | <0.0001 | ns | ns | ns |
| 25 vs. 50 | ns | ns | ns | ns | ns |
| 25 vs. 100 | ns | 0.0004 | ns | ns | ns |
| 50 vs. 100 | ns | ns | ns | ns | ns |
|  |  |  |  |  |  |
| **T_3_** |  |  |  |  |  |
| 0 vs. 6.25 | ns | <0.0001 | ns | ns | ns |
| 0 vs. 12.5 | ns | <0.0001 | ns | ns | ns |
| 0 vs. 25 | ns | <0.0001 | ns | ns | ns |
| 0 vs. 50 | ns | <0.0001 | ns | ns | ns |
| 0 vs. 100 | ns | <0.0001 | ns | ns | ns |
| 6.25 vs. 12.5 | ns | <0.0001 | ns | ns | ns |
| 6.25 vs. 25 | ns | <0.0001 | ns | ns | ns |
| 6.25 vs. 50 | ns | <0.0001 | ns | ns | ns |
| 6.25 vs. 100 | ns | <0.0001 | ns | ns | ns |
| 12.5 vs. 25 | ns | <0.0001 | ns | ns | ns |
| 12.5 vs. 50 | ns | <0.0001 | ns | ns | ns |
| 12.5 vs. 100 | ns | <0.0001 | ns | ns | ns |
| 25 vs. 50 | ns | ns | ns | ns | ns |
| 25 vs. 100 | ns | 0.0156 | ns | ns | ns |
| 50 vs. 100 | ns | ns | ns | ns | ns |
|  |  |  |  |  |  |
| **T_4_** |  |  |  |  |  |
| 0 vs. 6.25 | ns | 0.0004 | ns | ns | ns |
| 0 vs. 12.5 | ns | <0.0001 | ns | ns | ns |
| 0 vs. 25 | ns | <0.0001 | ns | ns | ns |
| 0 vs. 50 | ns | <0.0001 | ns | ns | ns |
| 0 vs. 100 | ns | <0.0001 | ns | ns | ns |
| 6.25 vs. 12.5 | ns | ns | ns | ns | ns |
| 6.25 vs. 25 | ns | <0.0001 | ns | ns | ns |
| 6.25 vs. 50 | ns | <0.0001 | ns | ns | ns |
| 6.25 vs. 100 | ns | <0.0001 | ns | ns | ns |
| 12.5 vs. 25 | ns | <0.0001 | ns | ns | ns |
| 12.5 vs. 50 | ns | <0.0001 | ns | ns | ns |
| 12.5 vs. 100 | ns | <0.0001 | ns | ns | ns |
| 25 vs. 50 | ns | 0.0156 | ns | ns | ns |
| 25 vs. 100 | ns | 0.0004 | ns | ns | ns |
| 50 vs. 100 | ns | ns | ns | ns | ns |
|  |  |  |  |  |  |
| **T_5_** |  |  |  |  |  |
| 0 vs. 6.25 | ns | ns | ns | ns | ns |
| 0 vs. 12.5 | ns | <0.0001 | ns | ns | ns |
| 0 vs. 25 | ns | <0.0001 | ns | ns | ns |
| 0 vs. 50 | ns | <0.0001 | ns | ns | ns |
| 0 vs. 100 | ns | <0.0001 | ns | ns | ns |
| 6.25 vs. 12.5 | ns | <0.0001 | ns | ns | ns |
| 6.25 vs. 25 | ns | <0.0001 | ns | ns | ns |
| 6.25 vs. 50 | ns | <0.0001 | ns | ns | ns |
| 6.25 vs. 100 | ns | <0.0001 | ns | ns | ns |
| 12.5 vs. 25 | ns | <0.0001 | ns | ns | ns |
| 12.5 vs. 50 | ns | <0.0001 | ns | ns | ns |
| 12.5 vs. 100 | ns | <0.0001 | ns | ns | ns |
| 25 vs. 50 | ns | ns | ns | ns | ns |
| 25 vs. 100 | ns | 0.0156 | ns | ns | ns |
| 50 vs. 100 | ns | 0.0156 | ns | ns | ns |
|  |  |  |  |  |  |
| **T_6_** |  |  |  |  |  |
| 0 vs. 6.25 | ns | ns | ns | ns | ns |
| 0 vs. 12.5 | ns | <0.0001 | ns | ns | ns |
| 0 vs. 25 | ns | <0.0001 | ns | ns | ns |
| 0 vs. 50 | ns | <0.0001 | ns | ns | ns |
| 0 vs. 100 | ns | <0.0001 | ns | ns | ns |
| 6.25 vs. 12.5 | ns | <0.0001 | ns | ns | ns |
| 6.25 vs. 25 | ns | <0.0001 | ns | ns | ns |
| 6.25 vs. 50 | ns | <0.0001 | ns | ns | ns |
| 6.25 vs. 100 | ns | <0.0001 | ns | ns | ns |
| 12.5 vs. 25 | ns | <0.0001 | ns | ns | ns |
| 12.5 vs. 50 | ns | <0.0001 | ns | ns | ns |
| 12.5 vs. 100 | ns | <0.0001 | ns | ns | ns |
| 25 vs. 50 | ns | ns | ns | ns | ns |
| 25 vs. 100 | ns | 0.0156 | ns | ns | ns |
| 50 vs. 100 | ns | 0.0156 | ns | ns | ns |

**Supplementary Table S6.** Data of expression levels in nauplii exposed to W + nZVI, W + PAHs, W + PAHs + nZVI, W + SED1 + nZVI and W + SED2 + nZVI were reported as a fold difference (in red up-expressed genes; in green down-expressed genes) from control (represented by adults of crustaceans reared in SW without contaminants) for 48 h. Fold differences greater than ± 1.5 were considered significant.

|  |  | **W + nZVI** | **W + PAHs** | **W + PAHs + nZVI** | **W + SED1 + nZVI** | **W + SED2 + nZVI** |
| --- | --- | --- | --- | --- | --- | --- |
| ***Stress*** | *hsp26* | 0.87 | -8.71 | -4.10 | -3.32 | -6.69 |
|  | *hsp60* | -1.64 | -3.72 | 2.16 | -8.13 | -1.78 |
|  | *hsp70* | 0.44 | 4.02 | 4.07 | 4.75 | 4.17 |
|  | *COXI* | -0.80 | -1.26 | 0.51 | 1.80 | -1.44 |
|  | *COXIII* | -3.14 | -3.53 | -3.56 | 2.38 | -3.90 |
| ***Development*** | *HAD* | -1.33 | 11.08 | -10.65 | -6.02 | -6.80 |
|  | *CDC48* | -1.26 | -5.78 | -7.47 | -6.65 | -7.30 |
|  | *tcp* | -0.82 | -1.91 | -0.79 | 1.94 | -0.38 |
|  | *UCP2* | 1.29 | 0.65 | 2.47 | 2.89 | 0.84 |

**Supplementary Table S7.** Data of expression levels in nauplii exposed to W + AC, W + PAHs, W + PAHs + AC, W + SED1 + AC and W + SED2 + AC, were reported as a fold difference (in red up-expressed genes; in green down-expressed genes) from control (represented by adults of crustaceans reared in SW without contaminants) for 48 h. Fold differences greater than ± 1.5 were considered significant.

|  |  | **W + AC** | **W + PAHs** | **W + PAHs + AC** | **W + SED1 + AC** | **W + SED2 + AC** |
| --- | --- | --- | --- | --- | --- | --- |
| ***Stress*** | *hsp26* | -0.69 | -8.71 | -0.53 | -0.01 | -0.27 |
|  | *hsp60* | 2.47 | -3.72 | 1.33 | 2.71 | 2.42 |
|  | *hsp70* | -0.05 | 4.02 | 3.42 | -1.69 | 4.58 |
|  | *COXI* | -0.07 | -1.26 | 2.37 | 2.71 | 2.70 |
|  | *COXIII* | -0.39 | -3.53 | 2.15 | 2.44 | 2.15 |
| ***Development*** | *HAD* | 0.51 | 11.08 | 0.39 | 1.84 | 3.60 |
|  | *CDC48* | 0.19 | -5.78 | 0.15 | 2.32 | 1.92 |
|  | *tcp* | 0.55 | -1.91 | 5.55 | 4.96 | 4.92 |
|  | *UCP2* | 0.62 | 0.65 | 3.65 | 3.74 | 2.88 |

**Supplementary Table S8.** Data of expression levels in adults exposed to W + nZVI, W + PAHs, W + PAHs + nZVI, W + SED1 + nZVI and W + SED2 + nZVI were reported as a fold difference (in red up-expressed genes; in green down-expressed genes) from control (represented by adults of crustaceans reared in SW without contaminants) for 48 h. Fold differences greater than ± 1.5 were considered significant.

|  |  | **W + nZVI** | **W + PAHs** | **W + PAHs + nZVI** | **W + SED1 +nZVI** | **W + SED2 + nZVI** |
| --- | --- | --- | --- | --- | --- | --- |
| ***Stress*** | *hsp26* | 7.52 | 13.73 | 7.42 | 7.07 | 6.92 |
|  | *hsp60* | 9.58 | 15.70 | 9.64 | 9.68 | 9.54 |
|  | *hsp70* | 2.67 | 5.93 | 1.71 | -2.52 | -7.68 |
|  | *COXI* | 2.69 | 10.00 | 5.56 | 4.46 | 3.79 |
|  | *COXIII* | 4.75 | 12.61 | 6.24 | 6.71 | 6.06 |

**Supplementary Table S9.** Data of expression levels in adults exposed to W + AC, W + PAHs, W + PAHs + AC, W + SED1 + AC and W + SED2 + AC, were reported as a fold difference (in red up-expressed genes; in green down-expressed genes) from control (represented by adults of crustaceans reared in SW without contaminants) for 48 h. Fold differences greater than ± 1.5 were considered significant.

|  |  | **W + AC** | **W + PAHs** | **W + PAHs + AC** | **W + SED1 + AC** | **W + SED2 + AC** |
| --- | --- | --- | --- | --- | --- | --- |
| ***Stress*** | *hsp26* | 1.09 | 13.73 | 13.73 | 17.48 | 10.72 |
|  | *hsp60* | 12.37 | 15.70 | 16.23 | 11.07 | 12.68 |
|  | *hsp70* | 1.25 | 5.93 | 7.61 | 5.65 | 5.33 |
|  | *COXI* | 1.10 | 10.00 | 12.19 | 10.69 | 11.69 |
|  | *COXIII* | 1.12 | 12.61 | 14.96 | 13.19 | 14.00 |

**Supplementary Figure S1.** Data of the sediment grain size analyzed with GradiStat software (version 8.0 based) to calculate particle size statistics for sieve or laser granulometric data. A) SED1; B) SED2.

**
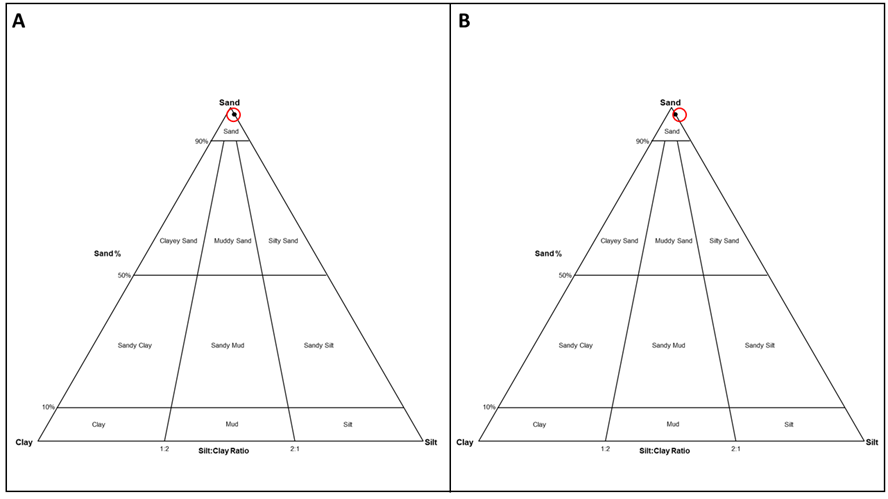
**

**Supplementary Figure S2.** A) Interactomic analysis by STRING (https://string-db.org/). The network graphically displays the relationship between genes. The biological relationships between genes are indicated by different colours. Known interactions: reported by database = light blue and determined experimentally = pink. Expected interactions: gene proximity= green; gene fusion = red; genes with similar pattern = light blue. B) Human gene names and the corresponding *A. franciscana* orthologous genes.

**
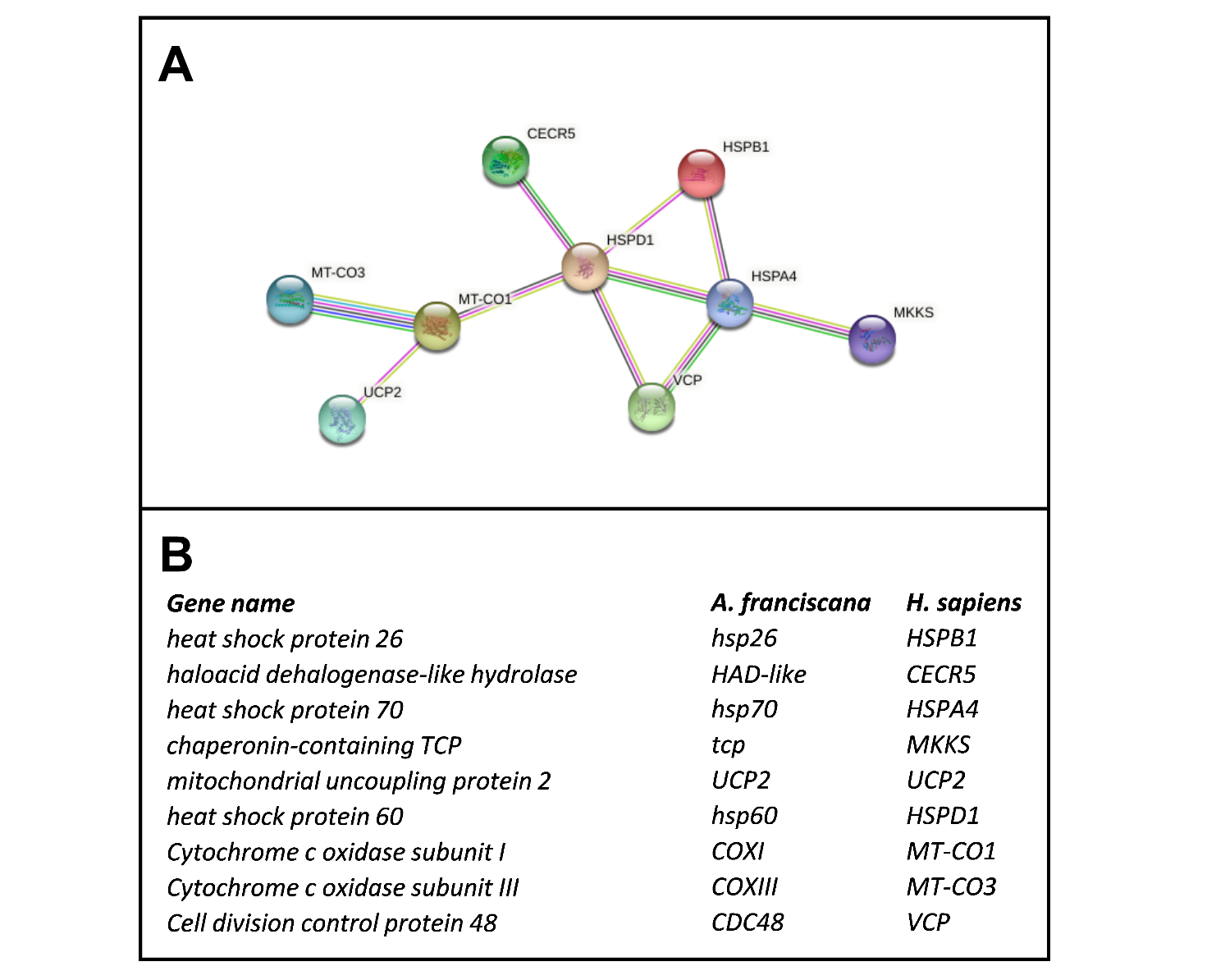
**

**Supplementary Figure S3.** Effect of nZVI dosage on PAHs removal. Experimental conditions: negative control (W + nZVI); positive control (W + PAHs); sediment spiked with PAHs + amendments (W + PAHs + nZVI); seawater + sediment1 (SED1) with amendments (W + SED1 nZVI); and seawater + sediment2 (SED2) plus amendments (W + SED2 + nZVI). Data points represent averages of three samples from triplicate reactors. (For more details regarding the removal of individual PAHs, see also **Supplementary** **Figure S4**).


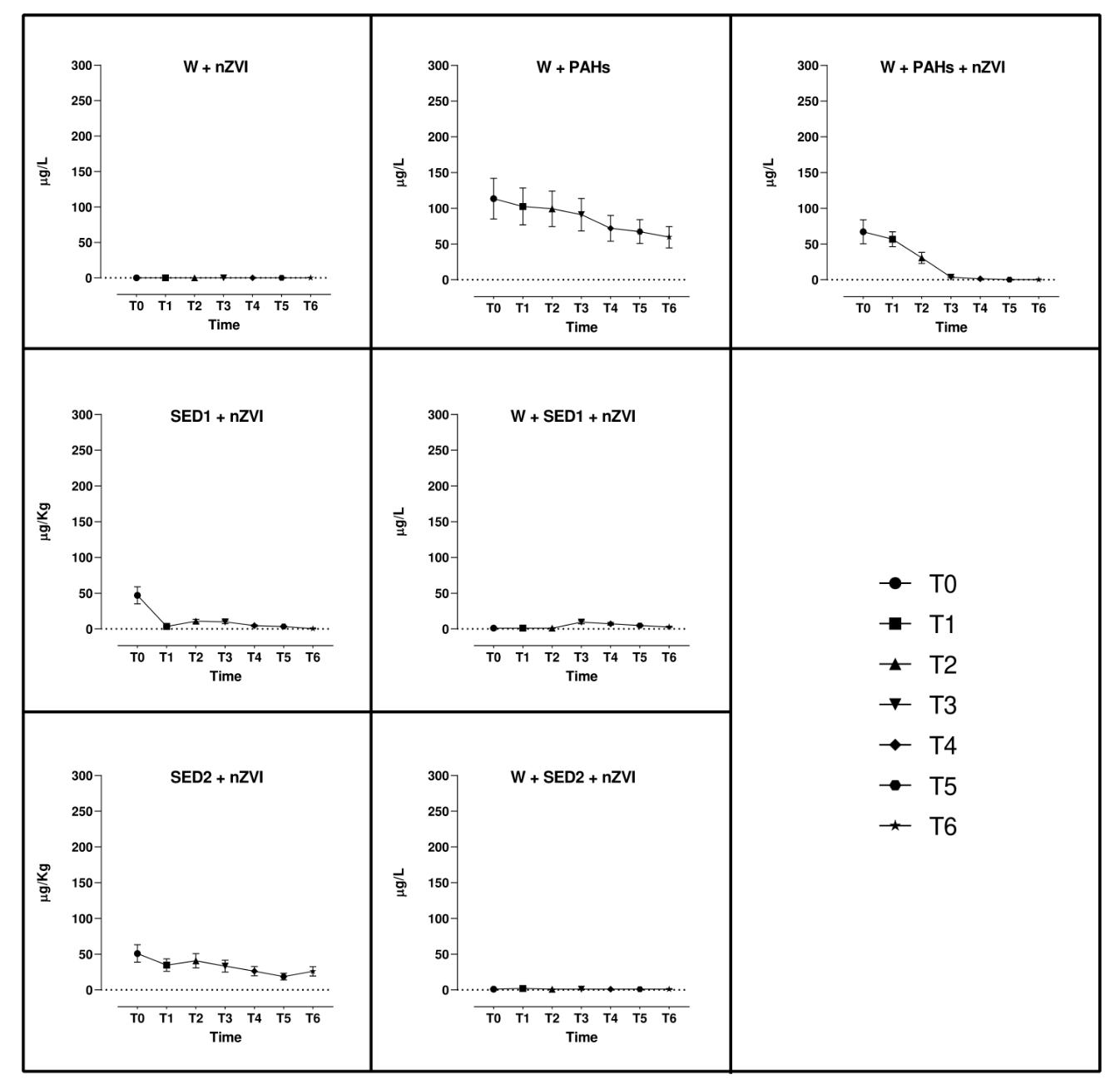


**Supplementary Figure S4.** Effect of nZVI dosage on single compound removal. Experimental conditions: negative control (W + nZVI); positive control (W + PAHs); sediment spiked with PAHs + amendments (W + PAHs + nZVI); seawater + sediment1 (SED1) with amendments (W + SED1 nZVI); and seawater + sediment2 (SED2) plus amendments (W + SED2 + nZVI). Data points represent averages of three samples from triplicate reactors.


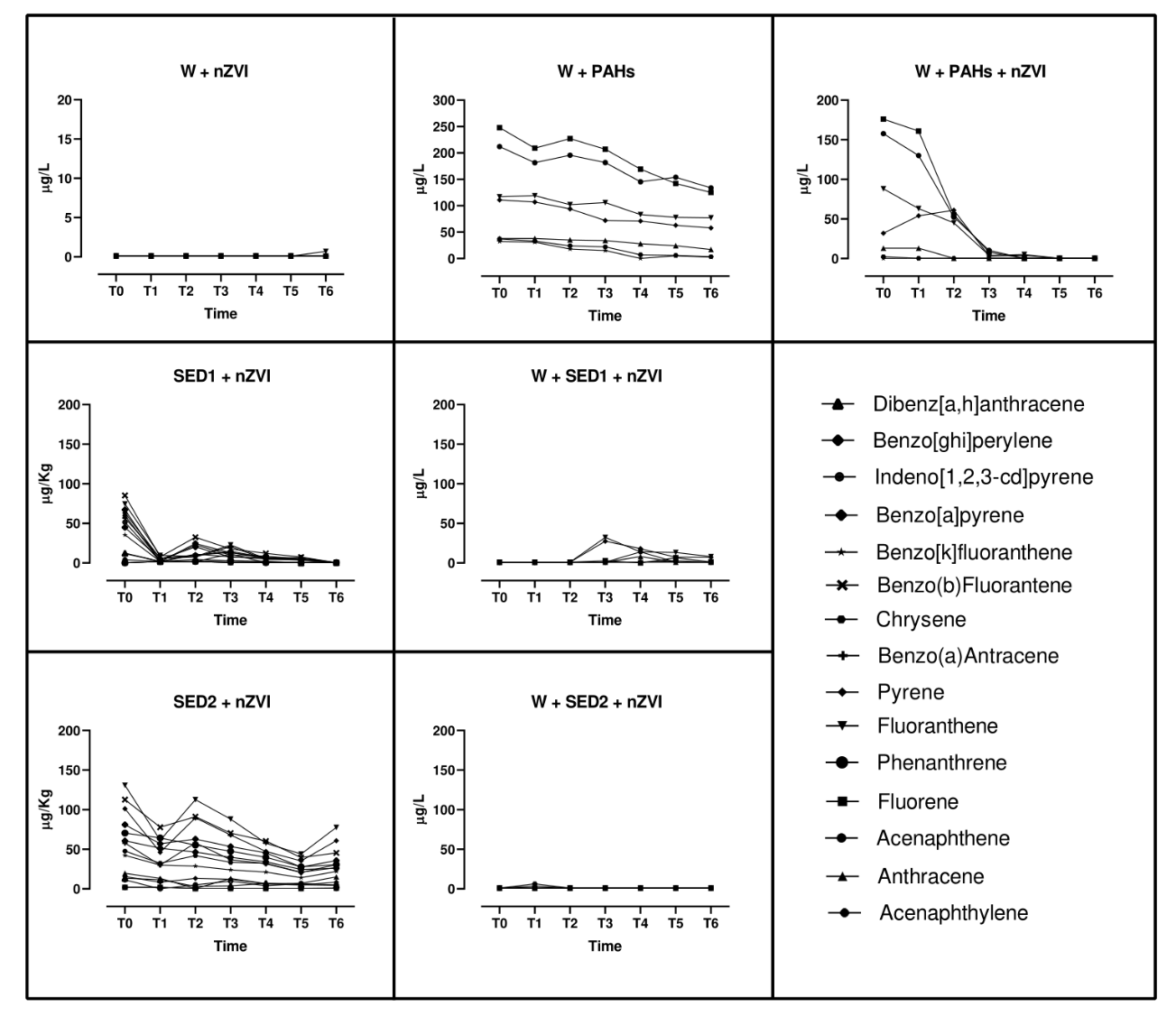


**Supplementary Figure S5.** Effect of AC dosage on PAHs removal. Experimental conditions: negative control (W + AC); positive control (W + PAHs); sediment spiked with PAHs + amendments (W + PAHs + AC); seawater + sediment1 (SED1) with amendments (W + SED1 + AC); and seawater + sediment2 (SED2) plus amendments (W + SED2 + AC). Data points represent averages of three samples from triplicate reactors. (For more details regarding the removal of individual PAHs, see also **Figure S6**).


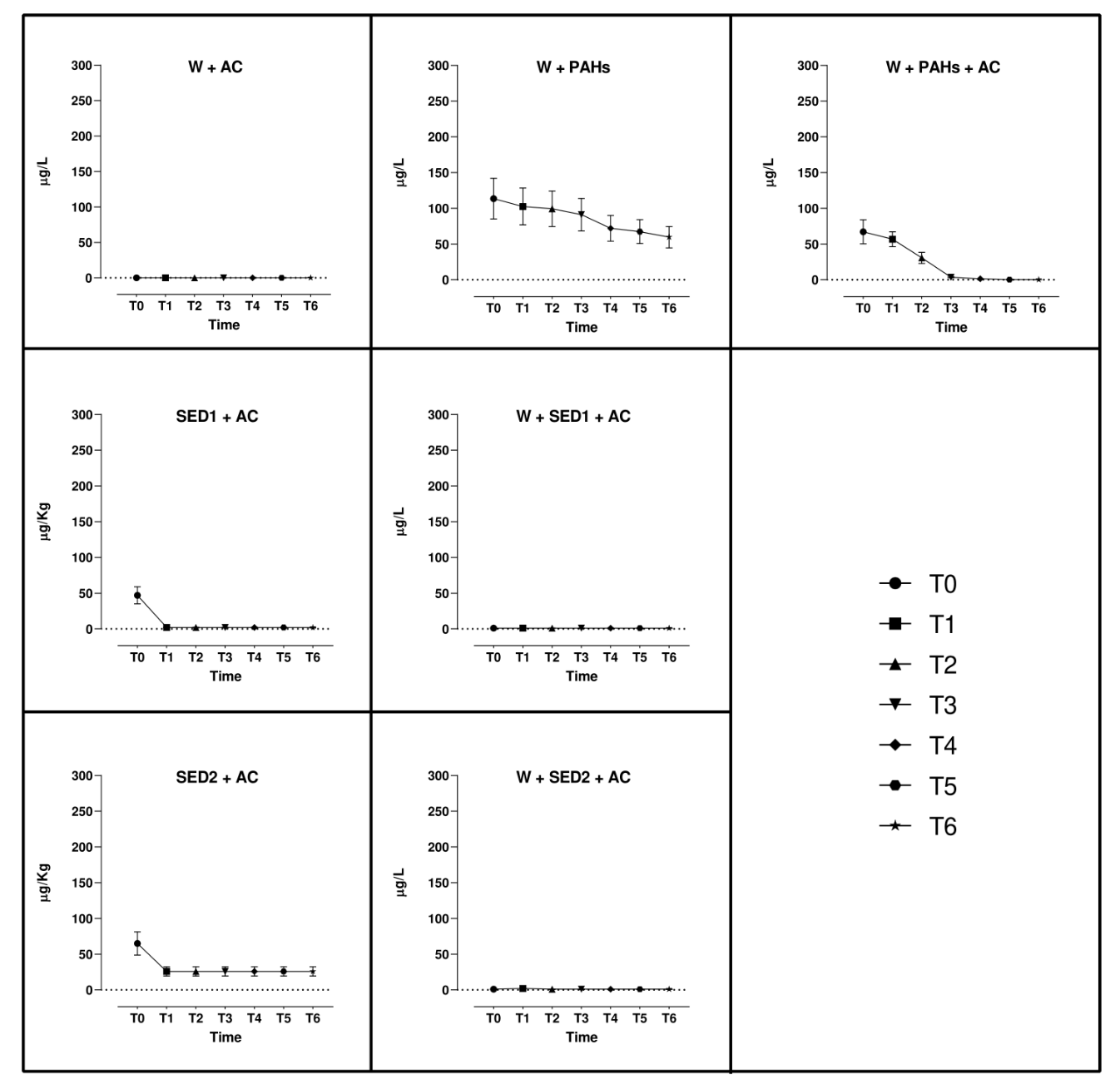


**Supplementary Figure S6.** Effect of AC dosage on single compound removal. Experimental conditions: negative control (W + AC); positive control (W + PAHs); sediment spiked with PAHs + amendments (W + PAHs + AC); seawater + sediment1 (SED1) with amendments (W + SED1 + AC); and seawater + sediment2 (SED2) plus amendments (W + SED2 + AC). Data points represent averages of three samples from triplicate reactors.


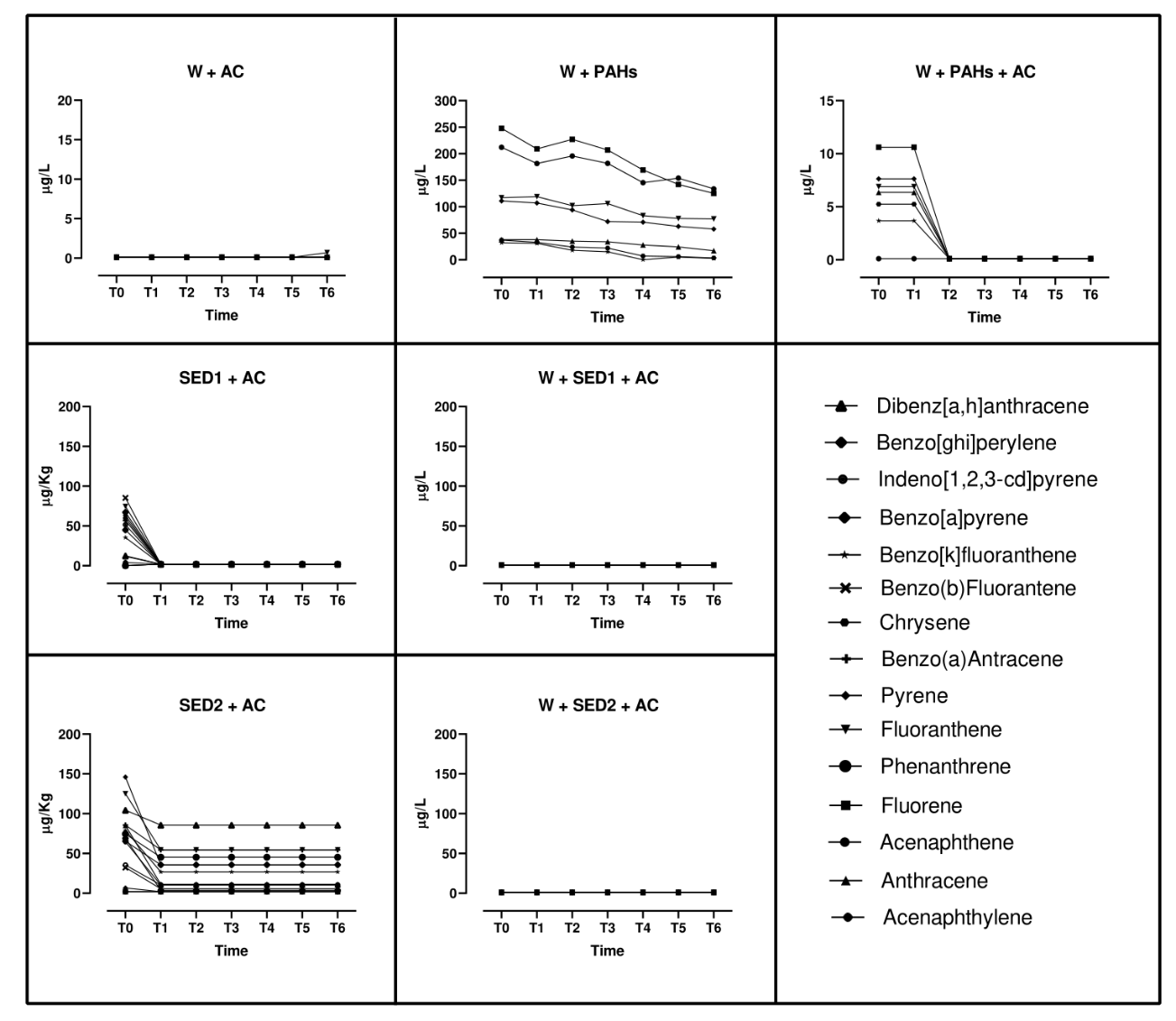

Supplement: Supplementary file 1 — Supplementary file1 (DOCX 2105 KB) [file 11356_2022_22408_MOESM1_ESM.docx]
